# Genetic and Biochemical Assays Reveal a Key Role for Replication Restart Proteins in Group II Intron Retrohoming

**DOI:** 10.1371/journal.pgen.1003469

**Published:** 2013-04-25

**Authors:** Jun Yao, David M. Truong, Alan M. Lambowitz

**Affiliations:** Institute for Cellular and Molecular Biology, Department of Chemistry and Biochemistry, and Section of Molecular Genetics and Microbiology, School of Biological Sciences, The University of Texas at Austin, Austin, Texas, United States of America; University of Michigan, United States of America

## Abstract

Mobile group II introns retrohome by an RNP-based mechanism in which the intron RNA reverse splices into a DNA site and is reverse transcribed by the associated intron-encoded protein. The resulting intron cDNA is then integrated into the genome by cellular mechanisms that have remained unclear. Here, we used an *Escherichia coli* genetic screen and Taqman qPCR assay that mitigate indirect effects to identify host factors that function in retrohoming. We then analyzed mutants identified in these and previous genetic screens by using a new biochemical assay that combines group II intron RNPs with cellular extracts to reconstitute the complete retrohoming reaction *in vitro*. The genetic and biochemical analyses indicate a retrohoming pathway involving degradation of the intron RNA template by a host RNase H and second-strand DNA synthesis by the host replicative DNA polymerase. Our results reveal ATP-dependent steps in both cDNA and second-strand synthesis and a surprising role for replication restart proteins in initiating second-strand synthesis in the absence of DNA replication. We also find an unsuspected requirement for host factors in initiating reverse transcription and a new RNA degradation pathway that suppresses retrohoming. Key features of the retrohoming mechanism may be used by human LINEs and other non-LTR-retrotransposons, which are related evolutionarily to mobile group II introns. Our findings highlight a new role for replication restart proteins, which function not only to repair DNA damage caused by mobile element insertion, but have also been co-opted to become an integral part of the group II intron retrohoming mechanism.

## Author Summary

Mobile group II introns are bacterial retrotransposons that are evolutionarily related to introns and retroelements in higher organisms. They spread within and between genomes by a mechanism termed “retrohoming” in which the intron RNA inserts directly into a DNA site and is reverse transcribed by an intron-encoded reverse transcriptase. The resulting intron cDNA is integrated into the genome by host factors, but how it occurs has remained unclear. Here, we investigated the function of host factors in retrohoming by genetic and biochemical approaches, including a new biochemical assay that reconstitutes the complete retrohoming reaction *in vitro*. Our results lead to a comprehensive model for retrohoming, which includes a surprising role for replication restart proteins in recruiting the host replicative DNA polymerase to copy the intron cDNA into the genome in the absence of DNA replication. We also find an unexpected contribution of host factors to initiating reverse transcription and a new RNA degradation pathway that suppresses retrohoming. We suggest that key features of the group II intron retrohoming mechanism may be used by human LINE elements and other non-LTR-retrotransposons. Additionally, our results provide new insights into the function of replication restart proteins, which are critical for surviving DNA damage in all organisms.

## Introduction

Mobile group II introns are non-long-terminal-repeat (non-LTR) retroelements that are commonly found in prokaryotes and in organellar genomes of eukaryotes and are thought to be evolutionary ancestors of splicesomal introns and retrotransposons in higher organisms [1]. They consist of an autocatalytic intron RNA (“ribozyme”) and an intron-encoded protein (IEP), which has reverse transcriptase (RT) activity. These two components function together in a ribonucleoprotein complex (RNP) to promote intron mobility by a mechanism in which the excised intron lariat RNA uses its ribozyme activity to reverse splice directly into a DNA site and is then reverse transcribed by the IEP, yielding an intron cDNA that is integrated into the genome by host enzymes [2]–[5]. By using this mechanism, group II introns insert at high frequency into specific DNA target sites in a process called “retrohoming” and at low frequency into ectopic sites that resemble the normal homing site in a process called “retrotransposition” or “ectopic retrohoming” [6]. These processes enabled the dispersal of group II introns to a wide variety of bacteria and some archaea and likely into eukaryotic nuclear genomes, where ancestral group II introns are thought to have evolved into both spliceosomal introns and non-LTR-retrotransposons [3], [7], [8].

Although the early reverse splicing and reverse transcription steps catalyzed by group II intron RNPs are common to retrohoming pathways in all organisms, the late host-mediated steps of second-strand DNA synthesis and cDNA integration can occur in different ways. In *Saccharomyces cerevisiae* mitochondria, where retrohoming was studied initially, cDNA integration occurs largely by a recombination mechanism in which the nascent intron cDNA initiated at the recipient allele invades an intron-containing allele for completion of intron DNA synthesis before switching back to the recipient DNA in the upstream exon [9], [10]. In bacteria, however, the fully reverse spliced intron RNA is reverse transcribed to yield a full-length intron cDNA that is integrated directly into the recipient DNA by a RecA-independent mechanism hypothesized to involve host DNA repair enzymes [5], [11], [12]. Recently, non-lariat, linear forms of the *Lactococcus lactis* Ll.LtrB intron RNA were found to retrohome in *Drosophila melanogaster* by using host non-homologous end-joining enzymes for cDNA integration [13]. However, host factors that function in late steps in the retrohoming of group II intron lariat RNAs, the major retrohoming pathway used in nature, have not been identified conclusively in any organism, and consequently, the mechanisms used for these steps have remained poorly understood.

**Figure 1:**
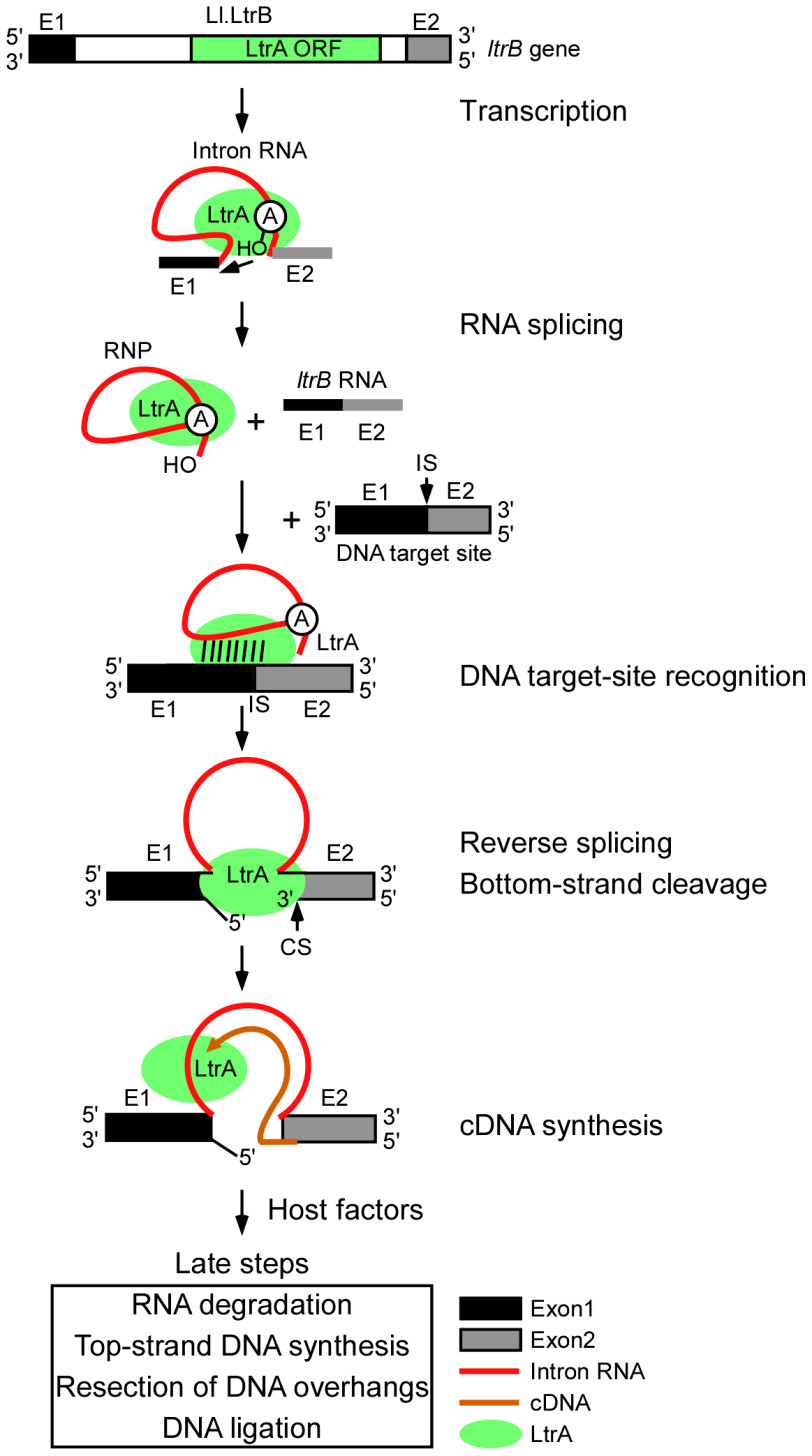
Retrohoming pathway of Ll.LtrB intron lariat RNA in bacteria. The Ll.LtrB intron, found in a relaxase gene (*ltrB*) in an *L. lactis* conjugative element, encodes a multi-functional RT (LtrA protein) with RT, RNA splicing, DNA-binding, and DNA endonuclease activities. Transcription of the *ltrB* gene yields a precursor RNA containing the intron flanked by 5′ and 3′ exons (E1 and E2, respectively). LtrA is translated from within the intron using its own Shine-Dalgarno sequence and then binds to the intron in the precursor RNA to promote formation of the catalytically active RNA structure for RNA splicing. RNA splicing occurs via two sequential RNA-catalyzed transesterification reactions that are initiated by nucleophilic attack of the 2′ OH of a branch point A-residue near the 3′ end of the intron at the 5′-splice site and results in ligated *ltrB* exons and an excised intron lariat RNA with a 2′-5′ phosphodiester linkage. After splicing, LtrA remains tightly bound to the excised intron lariat RNA in an RNP. RNPs initiate retrohoming by recognizing a DNA target site (the ligated *ltrB* E1–E2 sequence), using both the IEP and base pairing of the intron RNA. The intron RNA then inserts via reversal of the two transesterification reactions used for RNA splicing (referred to as “full reverse splicing”) into the intron-insertion site (IS) at the ligated-exon junction in the top strand of the DNA target site. LtrA uses its DNA endonuclease activity to cleave the bottom strand at a site (CS) between positions +9 and +10 of E2 and uses the 3′ DNA end at the cleavage site as a primer for reverse transcription of the inserted intron RNA. The resulting intron cDNA is then integrated into the genome by host enzymes in late steps that minimally include degradation of the intron RNA template strand, second (top)-strand DNA synthesis, resection of DNA overhangs, and sealing of DNA strand nicks [12].

Figure 1 diagrams the major steps elucidated thus far in the retrohoming pathway of the *L. lactis* Ll.LtrB intron, which has been studied extensively as a model system for group II intron lariat RNA retrohoming in bacteria. The Ll.LtrB intron was discovered in a relaxase gene (*ltrB*) in a conjugative element, where its splicing is required to produce functional relaxase for conjugation [14], [15]. Its IEP, denoted LtrA protein, is multifunctional, with RT, RNA splicing (“maturase”), DNA binding, and DNA endonuclease activities [16], [17]. Transcription of the *ltrB* gene yields a precursor RNA, which contains the intron flanked by the 5′ and 3′ *ltrB* exons (E1 and E2, respectively). The IEP, which is translated from within the intron, binds to the intron in the unspliced precursor RNA and promotes its splicing by stabilizing the catalytically active RNA structure [18]. Splicing occurs via two sequential RNA-catalyzed transesterification reactions that yield ligated *ltrB* exons and an excised intron lariat RNA to which the IEP remains tightly bound in an RNP. RNPs then initiate retrohoming by recognizing a DNA target sequence (corresponding to the ligated *ltrB* E1–E2 DNA sequence), using both the IEP and base pairing of the intron RNA [19]–[21]. After DNA target site recognition, the intron RNA fully reverse splices into the top strand of the DNA, leading to insertion of the intron RNA between the two DNA exons, while the IEP cleaves the bottom strand 9 nts downstream of the intron-insertion site and uses the 3′ end of the cleaved DNA strand for target DNA-primed reverse transcription (TPRT) of the inserted intron RNA [16], [17]. Finally, the resulting intron cDNA is integrated into the recipient DNA by host factors in late steps that minimally include the degradation or displacement of the intron RNA template strand, second (top)-strand DNA synthesis, resection of DNA overhangs, and ligation to seal DNA nicks [12].

In addition to its native host, the Ll.LtrB intron splices and retrohomes efficiently in a wide variety of other bacteria, including *Escherichia coli*, where it has been studied by using the facile genetic and biochemical methods available for that organism [5]. By screening *E. coli* mutants using two different plasmid-based retrohoming assays, we in collaboration with the Belfort laboratory previously identified candidate host factors that potentially function in the late steps in retrohoming, including RNase H1 and the 5′→3′ exonuclease activity of Pol I, both of which could contribute to degrading the intron RNA template strand; the host replicative polymerase Pol III, which may function in second-strand DNA synthesis; and DNA ligase A, which presumably seals strand nicks [12]. Decreased retrohoming frequencies were also found in mutants deficient in host exo- and endonucleases activities [RecJ, DnaQ (MutD), and SbcD], which could function to resect overhangs or resolve intermediates, and increased retrohoming frequencies were found for mutants deficient in RNases I and E and exonuclease III, which in wild-type strains may suppress retrohoming by degrading the intron RNA or nascent cDNA [12].

More recently, Coros et al. [22], [23] extended this work by screening an *E. coli* transposon-insertion library for mutants defective in group II intron retrohoming into chromosomal sites, using a donor plasmid to express an Ll.LtrB-ΔORF intron carrying a *kan^R^* marker. This screen identified additional host factors potentially involved in retrohoming, including polynucleotide phosphorylase (PNPase), the DNA helicase Rep, and MnmE (TrmE), which functions in tRNA modification, with additional host proteins (CyaA, SpoT, and AtpA) acting by affecting accessibility of chromosomal target sites or energy metabolism, and RNase E acting to impede retrohoming by degrading the intron RNA.

Although the genetic screens described above identified host genes in which mutations decrease retrohoming efficiency, it remains possible that some or many of these mutations affect retrohoming indirectly. Such indirect effects could result from mutations that impair the propagation or expression of the intron-donor plasmid, decrease the intracellular levels or activity of group II intron RNPs, or impede the accessibility of group II intron RNPs to DNA target sites. Additionally, all previous genetic screens relied upon the expression of an antibiotic-resistance marker to identify retrohoming events and are thus vulnerable to false positives arising from mutations that affect the expression of antibiotic-resistance (*e.g.*, by affecting the expression of the antibiotic-resistance gene or cellular permeability to the antibiotic). In some cases, studies of “indirect” effects revealed by the genetic screens have provided rich information about host responses and the regulation of group II intron mobility [22], [23]. However, further insight into the retrohoming mechanism requires the identification of host factors that function directly in this process.

Here we used an *E. coli* genetic screen and Taqman qPCR assay that mitigate indirect effects to identify candidate host factors for Ll.LtrB retrohoming, and we tested their function in retrohoming by using a newly developed biochemical assay that combines group II intron RNPs with cellular extracts to reconstitute the complete retrohoming reaction *in vitro*. By using these multiple approaches, we confirmed some previously identified host factors but not others. Additionally, we found that replication restart enzymes play a key role in intron retrohoming by initiating second-strand DNA synthesis. Our findings indicate a novel mechanism for the major pathway of group II intron lariat retrohoming in bacteria, with features that may be shared by human LINE elements and other non-LTR retrotransposons.

## Results

### Genetic and Taqman qPCR screens to identify host proteins that function in retrohoming

**Figure 2:**
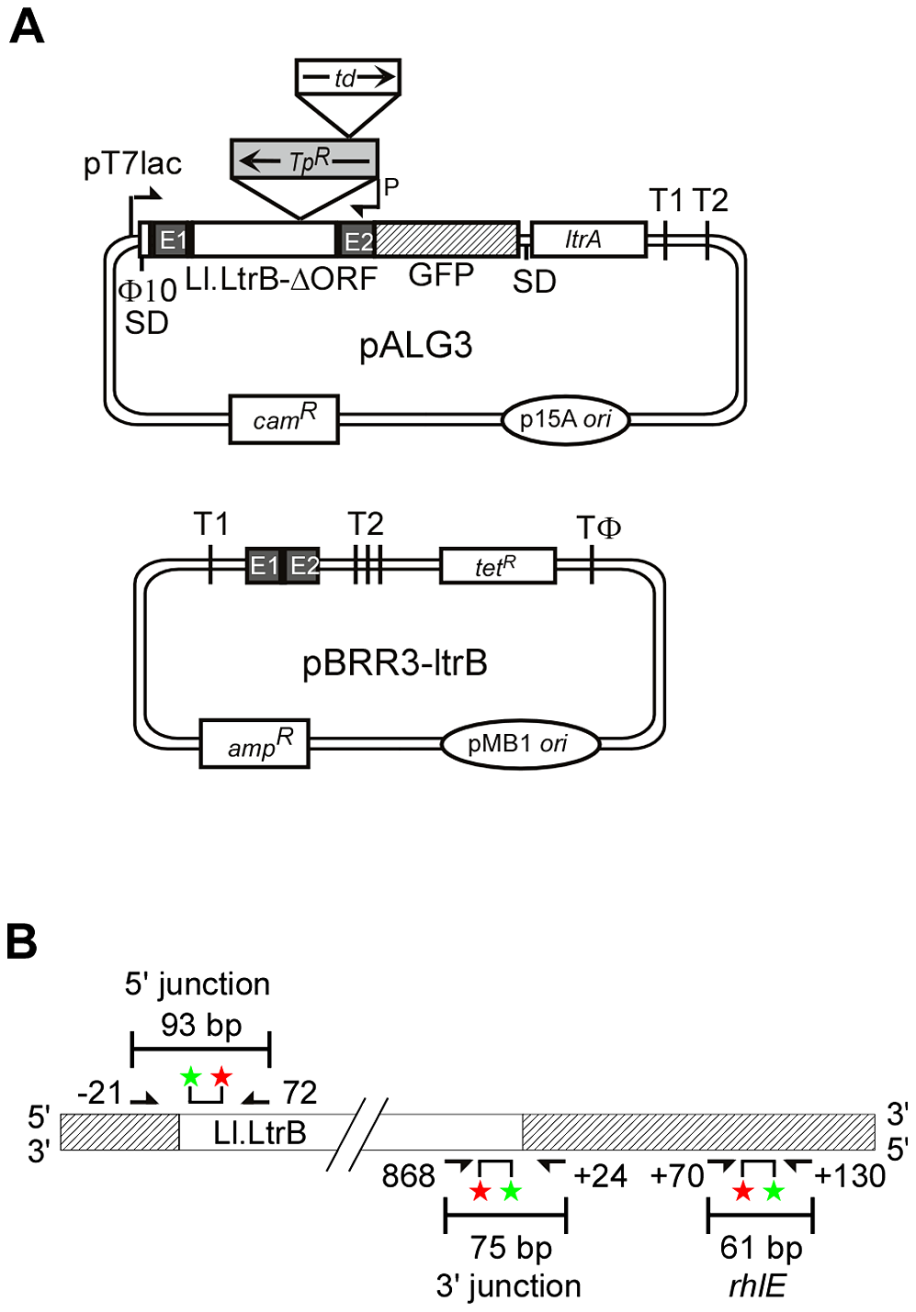
Genetic and Taqman qPCR assays used to identify *E. coli* mutants deficient in retrohoming. (A) Genetic assay. The Cam^R^ intron-donor plasmid pALG3 uses a T7lac promoter and phage Φ10 Shine-Dalgarno (SD) sequence to express an *ltrB*/GFP fusion cassette. This cassette consists of a 0.9-kb Ll.LtrB-ΔORF intron and flanking 5′- and 3′-exons (E1 and E2, respectively) [19], with the intron carrying a trimethoprim-resistance retrotransposition-activated genetic marker (Tp^R^-RAM), and E2 linked in-frame to an ORF encoding GFP. The LtrA ORF preceded by its own Shine-Dalgarno sequence is co-transcribed from a position downstream of the GFP ORF. The Amp^R^ recipient plasmid contains a 45-bp Ll.LtrB target site (ligated E1–E2 sequence) upstream of a promoterless *tet^R^* gene. T1 and T2 are *E. coli rrnB* transcription terminators, and TΦ is a phage T7 transcription terminator. (B) Taqman qPCR assay. The assay quantifies 5′- and 3′-intron integration junctions resulting from retrohoming of a retargeted Ll.LtrB-ΔORF intron into a site in the *rhlE* gene in the *E. coli* chromosome. Retrohoming events are quantified by Taqman qPCR, which utilizes the 5′→3′ exonuclease activity of Taq DNA polymerase to cleave a fluorescently labeled DNA probe that base pairs to an internal region of a PCR amplicon. Digestion of the probe by Taq DNA polymerase releases the FAM label (red star) free of the MGB quencher (green star), resulting in a quantifiable fluorescence signal for each amplification event. The numbers of 5′- and 3′-intron integration junctions relative to the number of *rhlE* targets were determined by quantifying the fluorescence signals in three separate PCRs relative to standard curves generated from serial dilutions of a reference plasmid (Materials and Methods). Primers for these PCRs are depicted by arrows with numbers indicating the positions of the 5′ nucleotide of the upstream primer and 3′ nucleotide of the downstream primer relative to the intron-integration junction.

To identify host factors that function in retrohoming of the Ll.LtrB group II intron, we used two complementary approaches that mitigate weaknesses of previous genetic screens. First, we used a plasmid-based group II intron retrohoming assay that controls for indirect effects to screen an *E. coli mariner* transposon-insertion library for mutants that have decreased or increased retrohoming efficiency. This screen used the intron-donor plasmid pALG3 and recipient plasmid pBRR3-ltrB (Figure 2A) and was done in *E. coli* host strain HMS174(DE3), which is RecA^−^ and encodes an isopropyl β-D-1 thiogalactopyranoside (IPTG)-inducible T7 RNA polymerase for donor-plasmid transcription. The donor plasmid pALG3, which was newly constructed for this screen, uses a T7lac promoter to synthesize a precursor RNA, which contains an Ll.LtrB-ΔORF intron (*i.e.*, an intron deleted for the LtrA ORF) flanked by short 5′ and 3′ exons, with the 3′ exon linked in frame to an ORF encoding GFP. The LtrA protein, which functions stoichiometrically to splice and mobilize the Ll.LtrB intron, is expressed from a position downstream of the GFP ORF, where it is co-transcribed with the intron RNA and translated using its own Shine-Dalgarno sequence. Because the expression of the Ll.LtrB-ΔORF intron and its splicing to produce RNPs are linked to GFP expression, mutants with decreased group II intron RNP production as a result of defects in donor plasmid replication, LtrA protein expression, or the expression and splicing of the Ll.LtrB intron RNA, are identified readily by decreased GFP fluorescence after IPTG induction.

To enable direct selection for retrohoming events, the Ll.LtrB intron in pALG3 carries a trimethoprim-resistance retrotransposition-activated genetic marker (Tp^R^-RAM), which consists of a small trimethoprim-resistance gene inserted in the orientation opposite group II intron transcription, but interrupted by an efficiently self-splicing group I intron, the phage T4 *td* intron, in the forward orientation [24]. During retrohoming via an RNA intermediate, the *td* intron is spliced, thereby reconstituting the Tp^R^ marker and enabling its expression after the intron retrohomes into the DNA target site. By using this combination of Tp^R^-RAM and GFP markers, mutants with transposon insertions that inhibit retrohoming without affecting Ll.LtrB expression or splicing are identified as Tp^S^ and GFP^+^.

The screen was done in 96-well plates under calibrated selective conditions in which the cell density for log phase cells provides a measure of retrohoming efficiency (Figure S1; Materials and Methods). Screening of 9,200 colonies by two rounds of 96-well plate assays identified 61 transposon insertions that reproducibly gave a >4-fold decrease in retrohoming efficiency compared to a wild-type control and had no defect in RNP production, as judged by a fluorescence-activated cell sorter (FACS) assay of GFP synthesis (Table S1). After mapping of transposon-insertion sites by thermal-asymmetric-interlaced (TAIL) PCR [25], we identified 67 candidate protein-encoding genes, whose disruption or altered expression due to the proximity of the transposon insertion results in decreased retrohoming efficiency. Six of these candidate genes were sites of multiple transposon insertions, and 12 were genes with nucleic acid-related functions found downstream of transposon-insertion sites within operons. An additional transposon insertion (C0719) that decreased retrohoming efficiency mapped to a site predicted to encode a small non-coding RNA (sRNA) (Table S1). All the candidates were confirmed by Southern hybridization to contain a single *mariner*-transposon insertion at the indicated genomic location (data not shown).

To complement the transposon-insertion screen, our second approach was to screen individual candidate strains for efficient integration into a chromosomal target site directly by using a Taqman qPCR assay to quantify both the 5′- and 3′-intron-integration junctions, thereby eliminating false positives that arise from mutations affecting expression of a drug-resistance phenotype (Figure 2B). Mobile group II introns can be retargeted to retrohome into different chromosomal DNA sites simply by modifying the intron RNA sequences that base pair to the DNA target sequence (see Introduction), a gene targeting technology known as “targetron” [19], [21], [26]. The Taqman qPCR assay uses an Ll.LtrB-ΔORF intron that was retargeted in this way to retrohome efficiently into a site in the *rhlE* gene, which encodes a non-essential DEAD-box protein whose disruption has no effect on cellular growth rate [21], [27]. The intron was expressed from the broad-host range donor plasmid pBL1, which has a different DNA replication origin than pALG3 and employs an *m*-toluic acid-inducible promoter; the latter functions independently of host factors and is activated by a freely permeable inducer (*m*-toluic acid) that does not require cellular transporters to enter the cell [28]. Additionally, the screen was carried out in mutant strains from the Keio collection in which deleted genes are replaced with a *kan^R^* marker, thereby mitigating polarity effects on downstream genes in operons [29]. The Keio strains were supplemented by temperature-sensitive mutants to test the contribution of essential genes.

We used the Taqman qPCR assay to test all 68 candidate host factors identified in our initial transposon-insertion screen, as well as 30 additional candidate proteins that act on nucleic acids, including all 21 such candidates identified in previous mutant screens [12], [22], [30]. Table 1 shows results of the Taqman qPCR assay for notable mutants, and Tables S1 and Table S2 show complete results for the *mariner*-transposon screen and Taqman qPCR assay, respectively. Among the 68 candidates identified in the initial transposon library screen (Table S1), only ten (*dnaC*, *dnaT*, *gyrB*, *mdoB*, *paoD*, *rpoH*, *rpoN*, *tonB*, *ydcM*, and *yjjB*) had statistically significant decreases in retrohoming efficiency in the Taqman qPCR assay, and only four (*dnaC*, *dnaT*, *gyrB*, and *rpoH*) had substantial decreases (10–67% of wild type retrohoming efficiency; Table 1 and Table S2). This poor correlation highlights the difficulty of distinguishing direct and indirect effects and the necessity of using multiple approaches to identify host factors that function in retrohoming.

**Table 1:** Taqman qPCR assays of retrohoming in notable *E. coli* mutants.

Gene	Function	Retrohoming frequency (% WT)	
		5′ Junction	3′ Junction
*dinB* †	DNA polymerase IV	125±4%	127±3%
*dnaB* ts	Replicative DNA helicase	41±13%	77±3%
*dnaC* ts ^,^ #	Replication/restart initiation	21±1%	10±1%
*dnaE* ts ^,^ †	DNA polymerase III, α-subunit	27±4%	47±9%
*dnaG* ts	DNA primase	36±5%	26±7%
*dnaQ* †	DNA polymerase III, ε-subunit	135±5%	128±5%
*dnaT* #	Primosomal protein I	39±3%	67±3%
*gyrB* ts ^,^ #	DNA gyrase, subunit B	14±8%	26±10%
*hns* †	Histone-like nucleoid structuring protein	20±5%	16±5%
*lexA* a	Transcriptional repressor of SOS	87±13%	81±13%
*ligA* ts ^,^ †	DNA ligase	113±1%	101±3%
*ligB*	DNA ligase	122±4%	102±4%
*mnmE* †	tRNA modification	128±12%	109±11%
*pnp* †	Polynucleotide phosphorylase	88±4%	102±1%
*polAex* ts ^,^ †	DNA polymerase I	38±5%	50±6%
*polB* †	DNA polymerase II	98±9%	84±7%
*priA*	Primosomal protein N′	52±2%	54±1%
*priB*	Primosomal protein N	92±1%	79±2%
*priC*	Primosomal protein N″	35±3%	23±3%
*recF* †	ss/dsDNA binding protein	125±6%	111±4%
*recJ* †	5′→3′ exonuclease	112±2%	104±5%
*recQ* †	ATP-dependent DNA helicase	115±7%	90±2%
*rep* †	ATP-dependent DNA helicase	167±5%	99±5%
*rnhA* †	RNase HI	11±1%	15±2%
*rnhB* †	RNase HII	106±3%	79±2%
*rpoH* #	RNA polymerase σ^32^ (σ^H^) factor	14±4%	11±2%
*sbcC* † ^,^ b	ATP-dependent dsDNA exonuclease	36±3%	31±1%
*sbcD* †	ATP-dependent dsDNA exonuclease	101±7%	103±8%
*seqA* †	Inhibitor of replication initiation	51±8%	44±7%
*ssb* ts	ssDNA binding protein	37±3%	41±1%
*stpA* †	H-NS-like DNA/RNA-binding protein	146±3%	123±5%
*tus* †	Inhibition of replication at Ter sites	39±8%	31±9%
*umuC* †	DNA polymerase V	119±3%	117±7%
*umuD* †	DNA polymerase V	142±5%	121±4%

The Table summarizes Taqman qPCR assays of Ll.LtrB intron retrohoming into a chromosomal *rhlE* target site in mutant strains. Keio deletion, non-temperature-sensitive mutants, and their parental wild-type strains containing donor plasmid pBL1-rhlE were grown to mid-log phase at 37°C and then intron expression was induced with 4 mM *m*-toluic acid for 1 h at 37°C. Five temperature-sensitive mutants (*dnaE^ts^*, *gyrB^ts^*, *ligA^ts^*, *rpoH^ts^*, and *ssb^ts^*) that could not be grown at 37°C and their parental wild-type strains were grown at 30°C and then shifted to 37°C for 1 h prior to a 1-h induction with 4 mM *m*-toluic acid at 37°C. The *priA* deletion strain was grown and induced at 30°C and was confirmed by sequencing to lack second-site suppressor mutations in *dnaC*, which are known to accumulate in PriA mutants [72]. Taqman qPCR was carried out on extracted DNA to determine the number of 5′- and 3′-integration junctions relative to the number of *rhlE* genes (see Figure 2B and Materials and Methods). Values are the mean ± S.E.M. for three experimental replicates normalized to the retrohoming frequency of the parental wild-type strain assayed in parallel. Mutants that showed decreased retrohoming frequencies were assayed at least three times. Retrohoming frequencies of parental wild-type control strains (for the mutants indicated in parentheses) expressed as percent of available *rhlE* targets sites were: BW25113 (Keio deletion strains) 34%; AB1157 (*dnaE^ts^*) 20%; N2603 (*ligA^ts^*) 40%; BW30384 (*polAex^ts^*) 25%; DG76 (*dnaB^ts^*, *dnaC^ts^*, and *dnaG^ts^*) 56%; KL921 (*ssb^ts^*) 40%; PR100 (*pnp^ts^*) 31%; SS996 (*ΔpriB*, *lexA*) 60%; EJ1261(*gyrB^ts^*) 30%; KY1445(*rpoH^ts^*) 30%.

ts Temperature sensitive.

† Genes in which mutations decreased retrohoming frequencies in published genetic screens [12], [22], [30].

# Genes also identified as contributing to retrohoming in the transposon-insertions screen using the Tp^R^-RAM assay in this work. Retrohoming efficiencies in the Tp^R^-RAM assay relative to the wild-type control were: *gyrB*, 1.1%; *recJ*, 18.9%: *rpoH*, 3.4%; and *yjjB* (upstream gene in operon with *dnaC* and *dnaT*), 0% (Table S1).

a The *lexA* mutant is *lexA51::Tn5* in the SS996 strain background and has a constitutively induced SOS response.

b The *sbcC* mutant was not deficient in retrohoming in Taqman qPCR assays of retrohoming at 30°C.

### Candidate host factors that function in retrohoming

Among the candidates identified as potential retrohoming factors in previous screens [12], [22], [30], the Taqman qPCR assay confirmed significant reductions in retrohoming efficiency in the Keio deletions of *rnhA* (RNase H1, the major cellular RNase H [31]); *seqA* (initiation of chromosomal DNA replication); *sbcC* (ATP-dependent exonuclease); *hns* (histone-like nucleoid structuring protein); and *tus* (DNA replication termination site-binding protein); as well as at restrictive temperatures in the temperature-sensitive mutants *polAex^ts^*, which is defective in the 5′→3′ exonuclease but not the DNA polymerase activity of Pol I [32]; and *dnaE^ts^* in the catalytic (α) subunit of the host replicative DNA polymerase Pol III [33]. Also in agreement with previous results, we found no strong decrease in retrohoming efficiency in a Keio deletion of *rnhB* (RNase H2 [34]).

In contrast to results of genetic assays, the Taqman qPCR assays found no decrease in retrohoming efficiency for Keio deletions of *recJ* (single-stranded DNA exonuclease [35]); *dnaQ* (Pol III ε subunit, which has the proofreading exonuclease activity [36]); *rep* and *recQ* (DNA helicases [37], [38]); *recF* (RecA-dependent recombination); *stpA* (H-NS-like DNA- and RNA-binding protein with RNA chaperone activity [39]); *mnmE* (*trmE*; tRNA modification); *ligA* and *ligB* (DNA ligases [40], [41]); and *pnp* (polynucleotide phosphorylase [42]). Additionally, the Taqman qPCR assay found no decrease in retrohoming efficiency for Keio deletions of the genes encoding DNA repair polymerases *polB* (Pol II [43]), *dinB* (Pol IV [44]), and *umuC* or *D* (Pol V [45]), whereas *polB* and *dinB* deletions in a different strain showed moderate decreases in previous genetic assays [12].

The new candidates that were identified in the transposon-insertion screen (Table S1) and confirmed to have substantial decreases in retrohoming efficiency in the Taqman qPCR assay (10–67% wild type; Table 1) were: DnaC and DnaT, which function in replication restart (identified as genes downstream of the transposon insertion in the *yjjB* operon [46], [47]); GyrB (DNA gyrase subunit B); and RpoH (RNA polymerase σ^32^ factor).

For several mutants in which inhibition of retrohoming was found in genetic assays but not in the Taqman qPCR assay, we subsequently found significant effects on top- or bottom-strand DNA synthesis in biochemical assays below (*e.g.*, *dinB*, *dnaQ*, *ligA*, *recJ*, *pnp*, *polB*, and *stpA*). The disagreement between the genetic and Taqman qPCR assays for these mutants may reflect: (i) that qPCR monitors only short DNA regions at the intron-integration junctions; (ii) the longer time of the Taqman qPCR assay, which may give alternative enzymes a greater chance to act; or (iii) the different genetic backgrounds of the strains used in the two assays. The results again emphasize the need to use multiple assays to identify retrohoming factors, with biochemical support for a genetic assay in our view providing the most definitive identification.

### Mutants deficient in group II intron RNP synthesis

The transposon-library screen in *E. coli* HMS174(DE3) identified eight retrohoming-deficient mutants that are Tp^S^/GFP^−^, potentially indicating a defect in the production of Ll.LtrB RNPs (Table S3). Although we hoped that such mutants would identify host factors required for Ll.LtrB intron splicing, all eight of these mutants have transposon insertions that likely affect donor plasmid transcription (four in the *lacUV5* promoter of the λDE3 prophage, one in the T7 RNA polymerase gene, and three in genes encoding membrane transporters that could affect IPTG induction or trimethoprim uptake: *ugpA* (glycerol-3-phosphate uptake transporter subunit); *xylF* (xylose transporter subunit); and *yjbB* (a putative transporter). Consistent with an effect on transcription, all eight mutants showed decreased GFP fluorescence when counter-screened with the control plasmid pALE, which lacks the Ll.LtrB intron and contains the ligated *ltrB* exons fused directly to GFP (Figure S2, Figure S3, and Table S3). A direct screen of the transposon library for splicing-defective mutants using plasmid pALG2, in which an Ll.LtrB-ΔORF intron lacking the Tp^R^-RAM marker is linked to GFP expression [48], also identified only mutants that are defective in GFP expression with both the reporter construct containing the intron and the control reporter construct lacking the intron [T7 RNA polymerase of the λDE3 prophage; *malE* (maltose ABC transporter subunit); *kdsD* (arabinose-5-phosphate isomerase); *dppD* (dipeptide ABC transporter subunit); *clcA* (H^+^/Cl^−^ exchange transporter); *yfeN* (conserved outer membrane protein); *pgi* (phosphoglucose isomerase; and *yjbE* (predicted protein)] (Table S4). These findings suggest either that splicing of the Ll.LtrB intron *in vivo* requires only the LtrA protein as it does *in vitro* [17] or that host-encoded splicing factors are essential proteins that are not readily identified in a transposon screen.

### Mutants with increased retrohoming efficiencies

The Tp^R^-RAM screen also identified five transposon insertions that give increased retrohoming efficiencies and potentially encode host factors that function to suppress retrohoming. Surprisingly, all five of these transposon-insertions mapped to three closely linked genes, *rnlA* (RNase LS), *yfjK* (DExH/D-box protein), and *yfjL* (protein of unknown function), which are part of a cryptic prophage (CP4-57) in *E. coli* K12 (Figure S4, Table S5; [49], [50]). In addition to higher group II intron retrohoming efficiencies indicated by high levels of Tp^R^, all five disruptants showed increased levels of GFP fluorescence both with the intron-donor plasmid pALG3, in which splicing of the Ll.LtrB-ΔORF intron is required for GFP expression, and with the control plasmid pALE, which has ligated *ltrB* exons fused directly to GFP (Figure S4). Given the identity of the affected genes, these findings suggest that the increased retrohoming efficiencies and GFP fluorescence result from decreased rates of RNA degradation, leading to elevated levels of group II intron RNPs and GFP mRNA. The degradosome, which functions in mRNA turnover in *E. coli*, is a multiprotein complex consisting of RNase E (an endoribonuclease), PNPase (an exoribonuclease), RhlB (a DEAD-box RNA helicase), and enolase [51]. The close linkage of the three genes potentially involved in intron RNA degradation in our screen suggests that they may function together in a previously unknown RNA degradation pathway, possibly a second degradosome.

### Replication restart proteins play a role in group II intron retrohoming

The decreased retrohoming efficiency resulting from a transposon insertion in the *yjjB* operon containing *dnaC* and *dnaT* in the Tp^R^-RAM screen (see above; Table S1) focused our attention on replication restart proteins as attractive candidates for playing a role in the late steps of retrohoming. We therefore carried out systematic Taqman qPCR assays of replication restart mutants and found significant reductions in retrohoming in Keio deletions of *priA*, *priC*, and *dnaT*, and in a temperature-sensitive mutant of *dnaB* (Table 1). PriA and PriC are key proteins that independently recognize stalled or collapsed replication forks in the three major *E. coli* replication restart pathways (denoted the PriA-PriB, PriA-PriC, and PriC-Rep pathways; [47], [52]), while DnaT interacts with PriA and PriC to load the replicative DNA helicase DnaB [53], [54]. We also found decreased retrohoming efficiencies in temperature-sensitive mutants of several essential genes that function in replication restart, including those encoding DnaC, which interacts with DnaB prior to loading [55]; DnaG, the DNA primase [56]; and the single-stranded DNA binding protein Ssb, which has been shown to promote the formation of the primosome at the chromosomal replication origin (*oriC*) and interacts with PriA to stimulate the loading of DnaB during replication restart [57], [58]. However, deletion of the genes encoding PriB, an auxiliary component of the PriA-dependent pathway, and Rep, which functions in conjunction with PriC [59], showed only small (1–21%) reductions in retrohoming efficiency.

We also carried out Taqman qPCR assays of retrohoming in a different set of replication restart mutants in the genetic background of *E. coli* SS996, a *recA* ^+^ strain containing a GFP reporter for SOS induction. The results indicated that the decreased retrohoming efficiencies in the affected replication restart mutants are not allele specific and are larger than can be accounted for by the proportion of cells undergoing the SOS response (Figure S5; different alleles tested for all genes except *dnaE* and *dnaB*). Additionally, the mutant strain SS4610 (*lexA51::Tn5*), which has a *lexA* null allele and is constitutively induced for the SOS response in nearly 100% of cells [60], showed only minimally decreased retrohoming frequencies in the Taqman qPCR assay (81–87% wild type; Figure S5 and Table 1). Collectively, the above findings indicate that the decreased retrohoming efficiency in the replication restart mutants is not a secondary effect of cell cycle arrest during SOS induction and indicate a requirement for replication restart proteins in group II intron retrohoming.

### An *E. coli* extract assay that reconstitutes retrohoming *in vitro*

**Figure 3:**
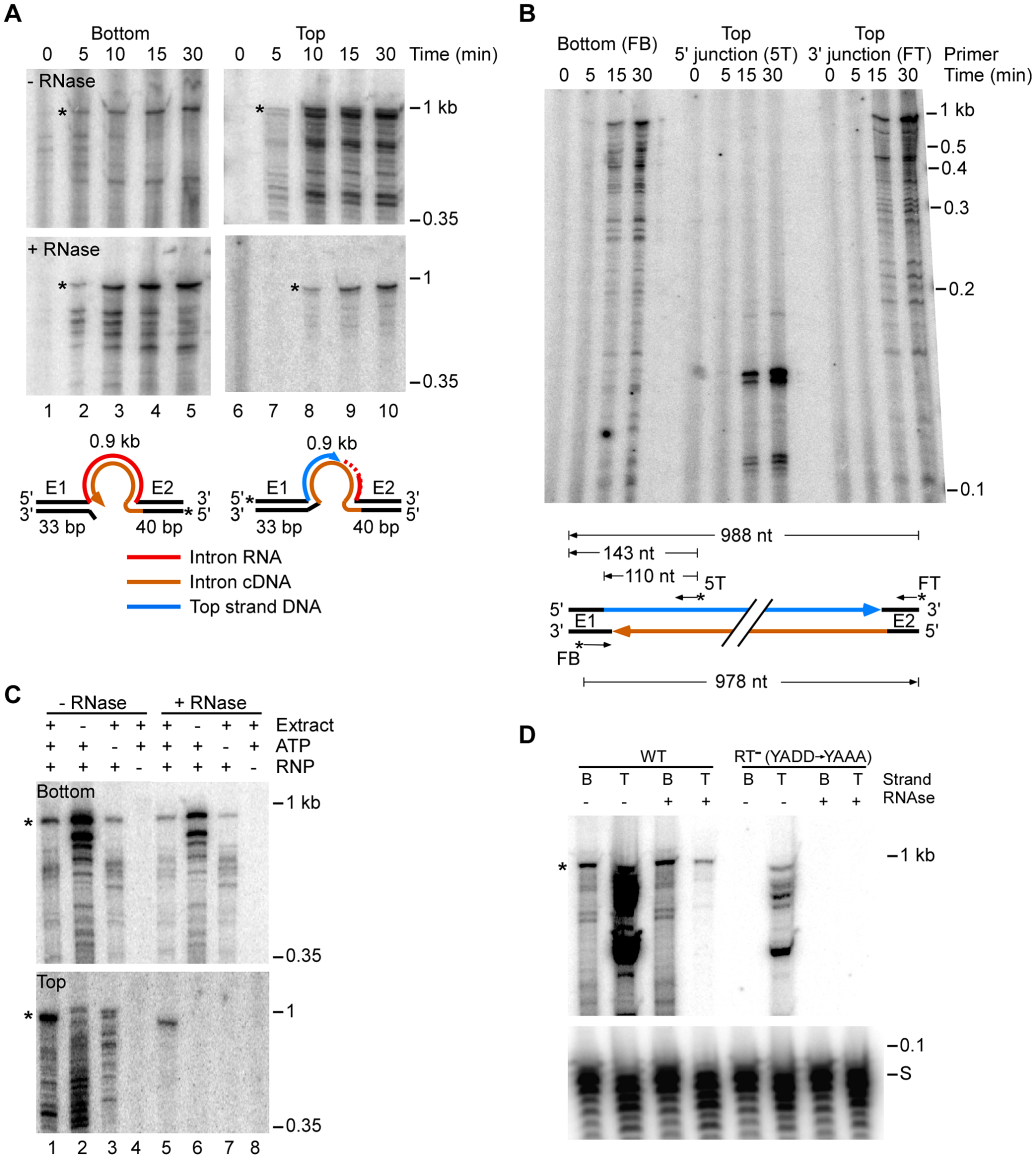
*E. coli* extract assay for bottom-strand (cDNA) and top-strand DNA synthesis. (A) Time courses. Group II intron RNPs and labeled DNA substrates (73 bp) containing the Ll.LtrB-insertion site (ligated E1–E2 sequence) were incubated with *E. coli* HMS174(DE3) extract in the presence of 1 mM dNTPs, 1.5 mM ATP, and an ATP-regenerating system (phosphoenolpyruvate+pyruvate kinase) at 37°C. The DNA substrates were labeled at the 5′ end of either the top (T) or bottom (B) strands to separately assay top- and bottom-strand DNA synthesis. After terminating portions of the reaction at the indicated times, samples were split into halves, which were incubated without or with RNases A+H, and the products were analyzed in a denaturing 6% polyacrylamide gel, which was dried and scanned with a PhosphorImager. RNase-sensitive top-strand products contain the reverse-spliced intron RNA. Schematics below the gels depict bottom- and top-strand synthesis on the DNA substrates (intron and exons not drawn to scale; star indicates 5′ ^32^P-label). (B) Primer extension analysis. DNA products synthesized in a time course were digested with RNase A+H, purified in a 1% agarose gel (0.85–1.2 kb gel slice), and analyzed by primer extension using 5′ -labeled primers to detect bottom-strand cDNAs (primer FB); the top-strand 5′-intron-integration junction (primer 5T); and top-strand DNAs (primer FT). Major products are diagrammed below the gel. (C) Requirements for bottom- and top-strand DNA synthesis. Reactions with the indicated components were incubated at 37°C for 30 min and then processed and analyzed in a denaturing 6% polyacrylamide gel, as described above. (D) Bottom- and top-strand products obtained with RNPs containing wild-type LtrA protein or an RT-deficient mutant LtrA (RT^−^; YADD motif changed to YAAA). For simplicity, the bottom part of the gel with the labeled DNA substrate (S) is shown only for panel D. Asterisks indicate gels bands of the size expected for full-length bottom- and top-strand products.

To further test the function of individual host proteins, we developed a biochemical assay in which host factors function together with group II intron RNPs to reconstitute the complete retrohoming reaction *in vitro*. This assay uses an *E. coli* S12 extract similar to those used for *in vitro* transcription and translation [61], [62]. Figure 3 shows experiments in which 5′ top- or bottom-strand labeled, 73-bp DNA oligonucleotide substrates containing the Ll.LtrB target site were incubated with group II intron RNPs in the presence of the extract, dNTPs (dATP, dCTP, dGTP, and dTTP), ATP, and an ATP-regenerating system (phosphoenolpyruvate+pyruvate kinase) at 37°C. The products were then analyzed in a denaturing polyacrylamide gel before and after digestion with RNases A+H to degrade the reverse spliced intron RNA leaving only 5′-labeled DNA products.

Time courses with the 5′-labeled bottom-strand substrate, which monitors cDNA synthesis, showed that an RNase-resistant band of the size expected for full-length bottom strand (988 nt) appeared after 5 min and accumulated for up to 30 min, along with a series of smaller bands (Figure 3A). These smaller bands are likely incomplete cDNAs rather than degradation products, as controls showed that exogenous ^32^P-labeled ssDNA and dsDNA corresponding to the retrohoming products were not degraded when incubated in the extracts under the same conditions (Figure S6).

Time courses with the 5′-labeled top-strand substrate, which monitors second-strand DNA synthesis, showed that an RNase-resistant band of the size expected for full-length top strand appeared later (10 min) and continued to accumulate during the reaction (Figure 3A, bottom right panel). We confirmed by primer extension that both the 5′- and 3′-junctions in the newly synthesized top strand DNA are continuous (Figure 3B).

Figure 3C shows that the appearance of labeled top- and bottom-strand products is dependent upon the addition of RNPs (lanes 4 and 8) and that top-strand DNA synthesis is completely dependent upon the presence of extract (lane 6). ATP increased the levels of reverse splicing and cDNA synthesis and was required for top-strand DNA synthesis in the extracts (*cf.*, lane 1 and 5 with lanes 3 and 7), indicating that energy-dependent processes, perhaps involving DNA or RNA helicases, are involved at both stages of the reaction. Further, RNPs containing a mutant LtrA protein that lacks RT activity (RT^−^; YADD→YAAA) carried out reverse splicing, but showed no detectable cDNA synthesis, indicating that bottom-strand synthesis is dependent upon the group II intron RT activity and is not done by a host polymerase in the extracts (Figure 3D).

### Biochemical analysis of retrohoming-deficient mutants

**Figure 4:**
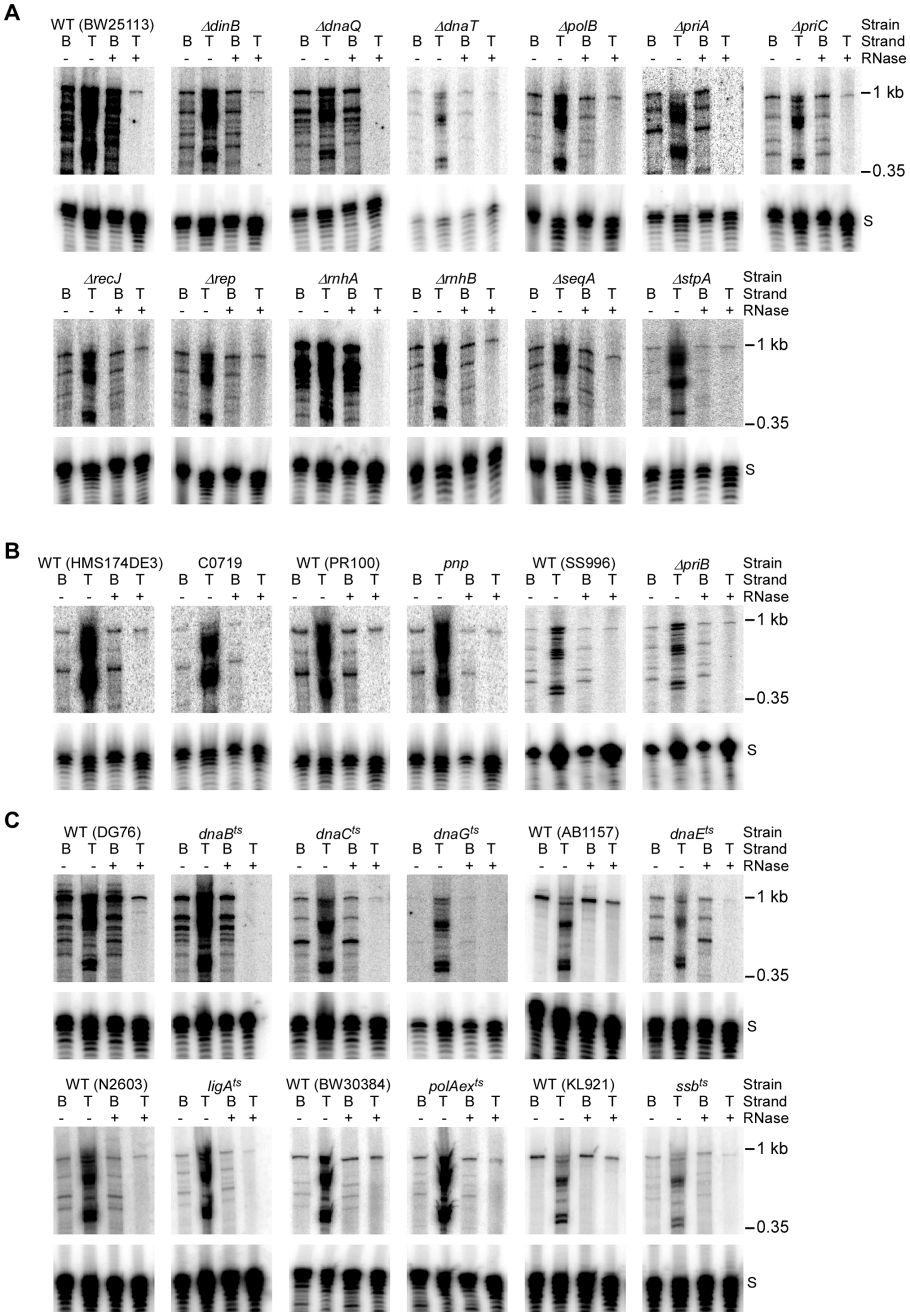
Assays of top- and bottom-strand DNA synthesis with extracts from *E. coli* mutants. DNA substrate labeled with ^32^P at the 5′-end of the top (T) or bottom (B) strand were incubated with group II intron RNPs for 15 min at 37°C in reaction medium containing extracts from: (A) Keio deletion mutants and their parental wild-type strain BW25113. (B) Mutant strain C0719, which contains a *mariner*-transposon at the site of a predicted sRNA, a *pnp* mutant, a *priB* deletion (non-Keio), and their parental wild-type strains; and (C) temperature-sensitive mutants and their parental wild-type strains. After phenol-CIA extraction and proteinase K digestion, samples were split into halves, which were incubated without or with RNase A+H at 37°C for 30 min. The products were analyzed in a denaturing 6% polyacrylamide gel, which was dried and scanned with a PhosphorImager. Extracts were confirmed to contain equal amounts of protein by SDS-polyacrylamide gels stained with Coomassie blue (not shown). The amount of radiolabel in the indicated product band or bands was normalized for the amount of substrate (S) in each lane and expressed as a percent of that in the parental wild-type strain (Table 2). At least two assays were done for each mutant and were reproducible to within <30%.

Finally, the *E. coli* extract assay enabled us to directly analyze bottom- and top-strand DNA synthesis in retrohoming-deficient mutants identified in genetic screens. In these experiments, extracts from Keio deletion or temperature-sensitive mutants were compared with those from their parental wild-type strains. In most cases, extracts were prepared from cells grown continuously at 37°C, which is a semi-permissive temperature for most of the temperature-sensitive mutants. The *priA* deletion strain was grown at 30°C to avoid accumulation of suppressor mutations, and five temperature-sensitive mutants (*dnaE^ts^*, *gyrB^ts^*, *ligA^ts^*, *rpoH^ts^*, and *ssb^ts^*) that could not grow at 37°C along with their parental wild-type strains were grown at 30°C and then shifted to 37°C for 2 h before preparing the extracts. For all strains, the extract assays were done at 37°C for relatively short times (15 min) to remain within the linear range and minimize dephosphorylation of the labeled DNA substrate for several mutants whose extracts appear to have elevated phosphatase activity (DnaQ, DnaT, PriA, and DnaB). Table 2 and Table S6 summarize quantitation of the assays, and representative assays for notable mutants are shown in Figure 4 and for the remaining mutants in Figure S7. All values shown in Table 2 and Table S6 were reproducible to within <30% in replicate experiments.

**Table 2:** *E. coli* extract assays of retrohoming in wild-type and mutant strains.

Strain	Reverse splicing	Total cDNA	Full-length bottom strand	Top strand
**Deletion and non-temperature sensitive mutants**				
BW25113	100%	100%	100%	100%
C0719^a^	57%	94%	69%	17%
*dinB*	66%	83%	32%	63%
*dnaQ*	78%	55%	98%	2%
*dnaT*	147%	95%	91%	50%
*hns*	165%	66%	50%	148%
*lexA* b	98%	81%	92%	121%
*ligB*	91%	101%	199%	147%
*mnmE*	90%	116%	103%	96%
*pnp* c	73%	109%	45%	42%
*polB*	137%	85%	43%	96%
*priA*	71%	70%	84%	1%
*ΔpriB* d	117%	123%	121%	116%
*priC*	105%	62%	33%	60%
*recF*	108%	94%	92%	87%
*recJ*	156%	101%	37%	98%
*recQ*	66%	71%	60%	78%
*rep*	112%	71%	49%	96%
*rnhA*	131%	143%	239%	2%
*rnhB*	138%	134%	218%	153%
*sbcC*	126%	82%	54%	104%
*sbcD*	89%	75%	64%	82%
*seqA*	80%	55%	76%	51%
*stpA*	81%	70%	23%	42%
*tus*	138%	83%	105%	166%
*umuC*	81%	91%	48%	89%
*umuD*	82%	74%	56%	78%
**Temperature-sensitive mutants**				
*dnaB^ts^*	170%	114%	62%	0%
*dnaC^ts^*	73%	75%	7%	18%
*dnaE^ts^*	46%	50%	7%	10%
*dnaG^ts^*	48%	4%	0%	0%
*gyrB^ts^*	93%	150%	215%	261%
*ligA^ts^*	100%	66%	15%	45%
*polA*ex*^ts^*	123%	112%	69%	34%
*rpoH^ts^*	84%	224%	166%	127%
*ssb^ts^*	85%	93%	24%	8%

Values were determined by measuring the amount of radioactivity in the indicated product band or bands relative to that in the substrate band after subtraction of background and are expressed relative to the corresponding values for the parental wild-type strain assayed in parallel. Total reverse splicing was quantified by measuring the radioactivity in all bands larger than the DNA substrate band in reactions with top-strand labeled DNA substrate without RNase treatment of the products. Total cDNA was quantified by measuring the radioactivity in all bands larger than the DNA substrate band in reactions with bottom-strand labeled DNA substrate after RNase treatment of the products. Full-length bottom strand was quantified by measuring the radioactivity in the band corresponding to the full-length bottom-strand product (988 nt) in reactions with 5′ bottom-strand labeled DNA substrate after RNase treatment of the products. Full-length top strand was quantified by measuring the radioactivity in the band corresponding to the full-length top-strand product (988 nt) after RNase treatment of the products. Radioactivity was determined by scanning the dried gel with a Typhoon Trio PhosphorImager and quantifying using ImageQuant TL.

a C0719 is a transposon-insertion mutant in the HMS174(DE3) strain background.

b The *lexA* mutant is *lexA51::Tn5* in the SS996 strain background and has a constitutively induced SOS response [60].

c The *pnp* mutant is the *pnp-7* allele in the PR100 strain background and has <10% wild- type PNPase activity [88].

d The *ΔpriB* mutation is a complete deletion of the gene in the SS996 strain background; the *priB* deletion strain sent by the Keio collection was found to retain the gene.

First, the biochemical assays confirmed the function of several candidate retrohoming factors that were expected to be required for top-strand DNA synthesis, including RNase H1 (*rnhA*), the 5′→3′ exonuclease activity of Pol I (*polAex^ts^*), and the replicative polymerase Pol III (*dnaQ*, *dnaE^ts^*). Extracts from Keio deletions or mutants having temperature-sensitive defects in these activities showed high levels of reverse splicing and cDNA synthesis, but strongly decreased top-strand DNA synthesis (*rnhA* and *dnaQ*, 2% wild type; *polAex^ts^*, 34% wild type; and *dnaE^ts^*, 10% wild type; Figure 4A, Figure 4C, and Table 2). The *ligA^ts^* mutant also showed a substantial decrease in top-strand DNA synthesis in the extract assays (45% wild type; Figure 4C), consistent with a role for DNA ligase A in sealing nicks. The incomplete inhibition of top-strand DNA synthesis in the temperature-sensitive mutants may reflect residual activity at the semi-permissive temperature used in the experiment. The Keio deletion of the second *E. coli* RNase H gene, *rnhB*, which had no effect on retrohoming *in vivo*, did not inhibit top-strand synthesis in the *in vitro* assays (153% wild type; Figure 4A). However, extracts from both the *rnhA* and *rnhB* deletions showed elevated levels of full-length intron cDNAs (239 and 218% wild type, respectively), possibly reflecting that RNase H2 makes some contribution to degrading the intron RNA template *in vitro*.

Among the replication restart mutants, we found substantial decreases in top-strand synthesis in extracts from Keio deletions *priA* (1% wild type), *priC* (60% wild type), and *dnaT* (50% wild type), as well as the temperature-sensitive mutants of *dnaB*, which encodes the replicative DNA helicase (0%), and *dnaC*, which interacts with DnaB prior to loading (18% wild type) (Figure 4A, Table 2). The greater effect of the Keio *priA* deletion *in vitro* than *in vivo* may reflect that the short time of the *in vitro* assays (15 min) favors intermediates with short gaps that are recognized by PriA, while the longer time of the *in vivo* assays (1 h) favors intermediates with longer gaps that are recognized by PriC [47]. In agreement with Taqman qPCR assays, we found no effect for the deletion of the genes encoding PriB, an accessory protein that facilitates PriA-DnaT complex formation (116% wild type; Figure 4B) [63], nor the DNA helicase Rep (96% wild type; Figure 4A), which ordinarily functions in conjunction with PriC [59].

We also found severe defects in top-strand DNA synthesis in the extracts from temperature-sensitive mutants of two essential proteins that function in replication restart pathways, the single-stranded DNA-binding protein Ssb (*ssb* ^ts^; 8% wild type) [57], [58] and the DNA primase (*dnaG^ts^*, 0%; [56]) (Figure 4C). Surprisingly, the DNA primase mutant (*dnaG^ts^*) was also strongly inhibited in bottom-strand cDNA synthesis (<1% wild type; Table 2; see Discussion). Importantly, the LexA SS4610 (*lexA51::Tn5*) mutant, which has a constitutively induced SOS response [60], showed no significant decrease in top- or bottom-strand DNA synthesis in the extract assays (92 and 121% wild type, respectively; Table 2, Figure S7), consistent with the minimal effect of this mutation on retrohoming *in vivo* (Table 1 and Figure S5).

Only four other protein mutants showed significantly decreased retrohoming in the extract assays (<70% wild-type top-strand synthesis): the Keio deletions of *dinB* (Pol IV; 63% wild type), *seqA* (51% wild type), and *stpA* (42% wild type; Figure 4A); and a *pnp* mutant that retains <10% of the wild-type PNPase activity (42% wild type; Figure 4B). Three of these mutants along with the Keio deletion of *polB* also showed substantially decreased synthesis of full-length bottom strands (*dinB*, 32% wild type; *pnp*, 45% wild type; *polB*, 43% wild type; and *stpA*, 23% wild type; Table 2). The effect of the DNA repair polymerase mutations is consistent with a possible role in helping to initiate at or traverse the intron RNA/DNA junctions in retrohoming intermediates [12] and/or in initiating replication restart before being replaced by Pol III [64]. PNPase, a component of the *E. coli* RNA degradosome, has multiple activities that could affect retrohoming, including 3′→5′ exoribonuclease, 3′-terminal oligonucleotide polymerase, high affinity binding to ssRNA and ssDNA, and lower affinity binding to dsDNA [65], [66]. StpA, an H-NS-like DNA- and RNA-binding protein that has RNA chaperone activity, could affect retrohoming by acting on either the target DNA or intron RNA [39], [67], [68]. How SeqA, a negative regulator of the initiation of chromosome replication [69], might contribute to retrohoming is unclear.

The RecJ deletion (single-stranded DNA exonuclease) is noteworthy for showing substantially decreased synthesis of full-length bottom strands (37% wild type; Figure 4A; Table 2) with no decrease in top-strand synthesis. RecJ mutants were identified as retrohoming-deficient both in initial genetic assays [12] and in the transposon screen in this work (Table 1), although not in the Taqman qPCR assays (see above). The effect of the *recJ* deletion on bottom-strand synthesis in the extract assays is consistent with its previously suggested function in resecting the 5′ overhang of the bottom-strand resulting from the staggered double-strand break made by group II intron RNPs [12].

Notably, we also found strong inhibition of top strand synthesis with extracts from the transposon-insertion mutant C0719, which is at the site of a predicted sRNA (17% wild type; Figure 4B and Table 2). Both reverse splicing and full-length cDNA synthesis were also decreased in this strain (57 and 69% wild type, respectively). How an sRNA might affect retrohoming warrants further investigation.

None of the other mutants tested strongly inhibited retrohoming in the *in vitro* assays (<70% wild type top-strand DNA synthesis; Figure 4, Table 2, Figure S7, and Table S6). A number of these mutants showed decreased retrohoming efficiencies in *in vivo* assays and may affect retrohoming indirectly by affecting chromosome structure, DNA replication, DNA target site accessibility, or energy production, *e.g.*, *gyrB^ts^* (DNA gyrase subunit B); *hns* (histone-like nucleoid structuring protein); *rpoH^ts^* and *rpoN* (RNA polymerase sigma factors); *tonB* (membrane protein involved in energy production); and *tus* (DNA termination site binding protein). Notably, the *gyrB^ts^* mutant showed decreased retrohoming efficiencies *in vivo*, but elevated levels of both top- and bottom-strand synthesis *in vitro* (261 and 215% wild type, respectively), possibly reflecting that DNA gyrase impedes retrohoming in wild-type extracts *in vitro* by unwinding some proportion of the dsDNA oligonucleotide substrate.

## Discussion

Here, we used genetic and biochemical approaches to identify *E. coli* host factors that function in group II intron retrohoming. First, we used a plasmid-based genetic assay that controls for indirect effects to screen a transposon-insertion library for mutants in which the Ll.LtrB group II intron shows decreased or increased retrohoming efficiency. We then used a Taqman qPCR assay to quantify retrohoming into a chromosomal site in Keio deletions or temperature-sensitive mutants of the candidates identified in this and previous screens. Finally, we compared retrohoming activity in wild-type and candidate mutant strains by using a new biochemical assay that combines Ll.LtrB RNPs with *E. coli* extracts to reconstitute the complete retrohoming reaction *in vitro*. Although the initial transposon screen remained vulnerable to false positives, it yielded a manageable group of candidates, whose function in retrohoming was verified by the more direct Taqman qPCR and/or biochemical assays.

**Figure 5:**
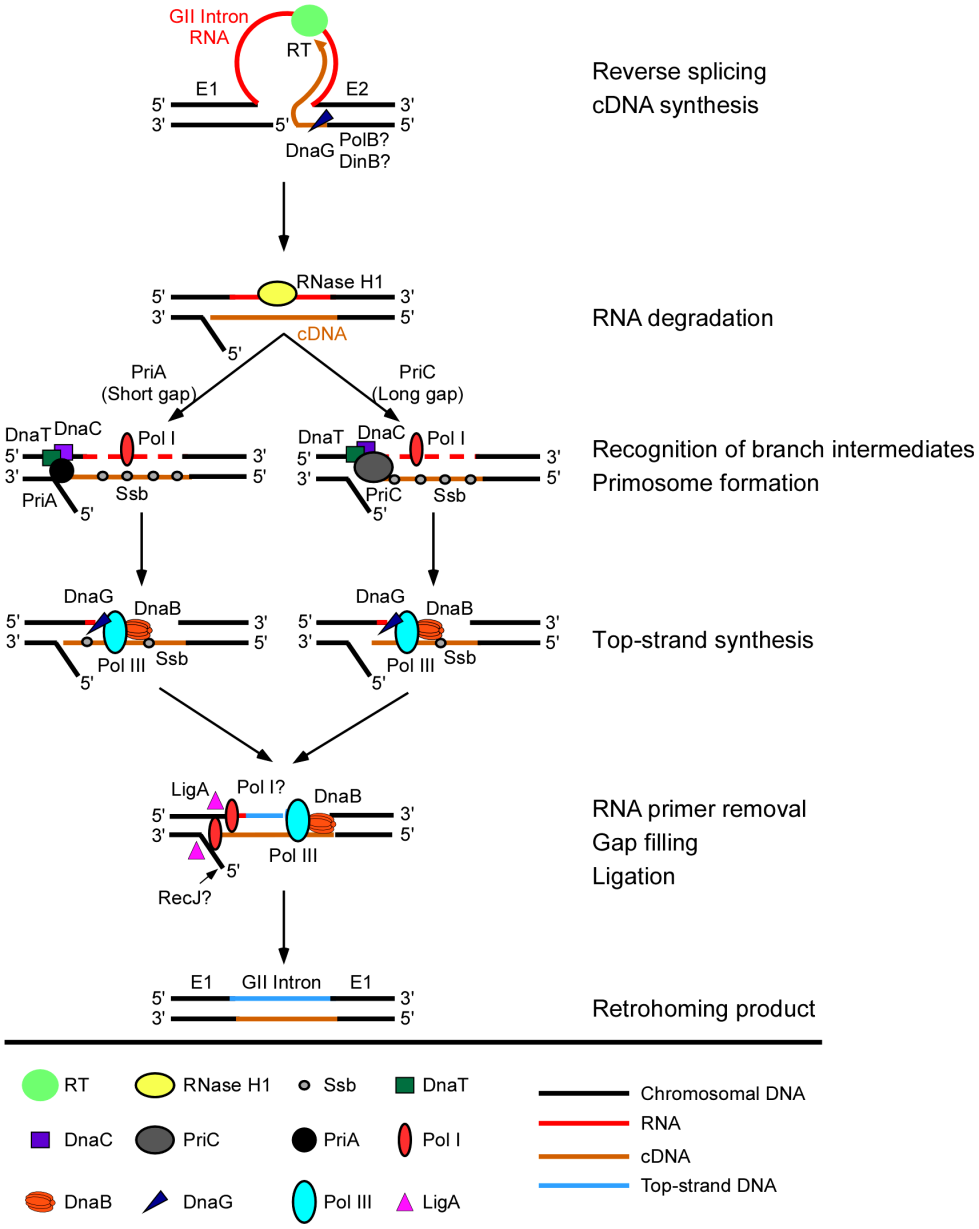
Model for function of host factors in group II intron retrohoming in *E. coli*. In initial steps, the group II intron lariat RNA reverse splices into the top strand of the DNA target site, while the intron-encoded RT cuts the bottom DNA strand and uses the 3′ end of the cleaved strand as a primer for target DNA-primed reverse transcription of the intron RNA. During or after cDNA synthesis, a host RNase H (RNase H1) degrades the intron RNA template strand. Extension of the intron cDNA into the 5′ exon displaces the bottom-DNA strand resulting in a branched intermediate that is recognized by the replication restart proteins PriA or PriC, with PriA preferentially recognizing intermediates with short gaps in the bottom strand and PriC preferentially recognizing intermediates with long gaps in the bottom strand. PriA and PriC then initiate a replisome loading cascade involving the sequential recruitment of the replicative helicase DnaB, the primase DnaG, and the replicative polymerase Pol III for second-strand DNA synthesis. Ssb stabilizes single-stranded DNA in gapped regions and interacts with PriA to stimulate the loading of DnaB. The 5′→3′ exonuclease activity of Pol I contributes to the removal of residual RNA primers and its DNA polymerase activity may contribute to filling in gaps, and a host DNA ligase (LigA) seals nicks in the top and bottom strands. Although bottom-strand synthesis is completely dependent on group II RT activity (Figure 3D), biochemical assays show that it is strongly inhibited in a DNA primase (DnaG) mutant and moderately inhibited in repair DNA polymerase DinB and PolB mutants, suggesting a previously unsuspected role for host factors in initiating bottom-strand (cDNA) synthesis. Deletion of RecJ moderately inhibits synthesis of full-length bottom strands in extracts, consistent with a role in resection of the 5′-overhang resulting from the staggered cleavage of the DNA substrate by group II intron RNPs [12].

Considered together, our results suggest a model for retrohoming of Ll.LtrB intron lariat RNAs in *E. coli* shown in Figure 5. In initial previously characterized steps, Ll.LtrB RNPs recognize the double-stranded DNA target site and the intron RNA reverse splices into one DNA strand, while the IEP cuts the opposite DNA strand and uses the cleaved strand as a primer for reverse transcription of the reverse-spliced intron RNA [5], [16]. The major host RNase H, RNase H1 encoded by *rnhA*, degrades the intron RNA template strand during or after cDNA synthesis, leaving residual RNA fragments that could serve as primers for top-strand DNA synthesis. Crucially, after synthesis of a full-length intron cDNA, either the group II intron RT or host DNA polymerase extends bottom-strand synthesis into the 5′ exon, yielding a branched intermediate that is recognized by the replication restart proteins PriA or PriC, which act preferentially on intermediates with short or long gaps between the branch and the 3′ end of the nascent strand [47]. These replication restart proteins then initiate a replisome-loading cascade leading to top-strand DNA synthesis by the host replicative polymerase, Pol III. We find that the 5′→3′ exonuclease activity of Pol I is required for second-strand synthesis during retrohoming, presumably to degrade RNA primers attached to newly synthesized DNA, and Pol I DNA polymerase activity could additionally contribute by helping to fill gaps, both functions of Pol I in host cell DNA replication [70], [71]. Surprisingly, although bottom-strand cDNA synthesis in the extracts is completely dependent upon the RT activity of the LtrA protein, it was nevertheless strongly inhibited in the DNA primase mutant *dnaG^ts^*, suggesting a previously unsuspected contribution of host factors to initiating cDNA synthesis (see below). The genetic screens also revealed a new putative *E. coli* RNA degradation pathway that impedes retrohoming and whose disruption leads to increased retrohoming efficiencies.

Importantly, our results confirm that the Ll.LtrB group II intron relies on host DNA polymerases for second-strand DNA synthesis, with a major role for the host replicative polymerase Pol III. The involvement of Pol III in second-strand DNA synthesis was postulated previously based on two findings: (i) that LtrA has low DNA-dependent DNA polymerase activity on artificial substrates *in vitro* [12], [13], and (ii) that DnaQ and DnaE^ts^ mutants are deficient in retrohoming in plasmid-based genetic assays [12]. Here, biochemical assays with cell extracts show that second-strand synthesis is completely dependent upon host DNA polymerases and is strongly inhibited in extracts from DnaQ and DnaE^ts^ mutants (2 and 10% wild-type activity, respectively). Notably, although on-going DNA replication may contribute to retrohoming *in vivo*, we observed the synthesis of a complete second-strand DNA *in vitro* in the absence of DNA replication.

A major function for RNase H1 in retrohoming is indicated by the findings that a Keio deletion and other mutations in the *rnhA* gene strongly inhibit retrohoming in genetic, Taqman qPCR, and biochemical assays in this work, and in two different genetic assays used in previous work [12]. By contrast, mutations in the *rnhB* gene encoding RNase H2 do not significantly inhibit retrohoming or top-strand synthesis in these assays (this work and [12]). Biochemical analysis using extracts from the Keio *rnhA* deletion strain show that deficiency of RNase H1 results in the accumulation of an intermediate containing the reverse spliced intron RNA, as expected, and that inability to degrade the intron RNA template strand strongly inhibits top-strand DNA synthesis. Extracts from the Keio *rnhB* deletion also showed some accumulation of the reverse-spliced intermediate, but no deficiency in top-strand synthesis.

A major finding is that replication restart proteins function in retrohoming and are required for second-strand DNA synthesis. We hypothesize that these proteins recognize the branched intermediate formed after RNase H degradation of the intron RNA template strand and extension of intron cDNA synthesis into the 5′ exon and then initiate replisome loading and second-strand DNA synthesis by Pol III by mechanisms similar or identical to those ordinarily used for replication restart at stalled or collapsed replication forks. The replication restart components found here to function in retrohoming by both *in vivo* and *in vitro* assays include PriA and PriC, the host proteins that initiate replication restart by recognizing stalled or collapsed replication forks [47]; the accessory proteins DnaC and DnaT [72]; and the replicative helicase DnaB [54]. Our biochemical assays with mutant extracts show directly that all these components are required for second-strand DNA synthesis. During replication restart, PriA recognizes a branched intermediate in which the 3′ OH of the nascent leading strand is close to the replication fork (no gap or a gap of <3 nts), while PriC recognizes an intermediate with a larger gap (>7 nts) [47]. During retrohoming, longer or shorter gaps in the branched intermediate could result from more or less resection of a stalled nascent bottom strand after dissociation of the RT prior to reinitiation of DNA synthesis by a host DNA polymerase. Although the top strand of the retrohoming intermediate contains annealed RNA fragments that result from RNase H digestion and may thus resemble a nascent lagging strand, the location of this strand relative to the branch differs from that at a replication fork, and it is unclear how or if it might also contribute to recognition by PriA or PriC.

The genetic and biochemical assays also indicate a major role in retrohoming for two other essential proteins that function in conjunction with replication restart machinery, the single-stranded DNA binding protein Ssb and the primase DnaG, with mutations in these proteins inhibiting both retrohoming *in vivo* and top-strand DNA synthesis *in vitro*. Ssb binds ssDNA regions after unwinding by Rep or PriA and has been shown to physically interact with PriA to stimulate the loading of DnaB at stalled forks [47], [58]. DnaG synthesizes short RNA primers, which are used for initiation of DNA synthesis by Pol III, and triggers the release of DnaC from DnaB [73]. The very stringent requirement for the helicase DnaB in the *in vitro* retrohoming reaction with a small DNA oligonucleotide substrate could reflect that in addition to DNA unwinding, it is needed to recruit the primase DnaG.

In contrast to other replication restart components, we found no contribution to group II intron retrohoming for PriB, an accessory protein in the PriA-PriB pathway, or Rep, which ordinarily functions together with PriC on the stalled fork by unwinding the dsDNA, especially when the 5′ end of the newly synthesized lagging strand is close to the fork [59], [74]. The dispensability of these factors presumably reflects that PriA can function independently of PriB in the PriA-PriC pathway and that PriC can load DnaB on stalled forks independently of either PriA or Rep [47], [59], [74]. Although the lack of requirement for Rep *in vitro* could also reflect that the biochemical assay uses a small DNA oligonucleotide substrate, it is nevertheless consistent with the lack of requirement for Rep in our *in vivo* assays (Table 1; in agreement with [12] but not [22]). An intriguing possibility is that a replisome assembled by replication restart proteins at the site of a group II intron insertion initiates a new round of host DNA replication from this location, thereby rapidly fixing the group II intron insertion into the genome.

The genetic and biochemical assays are consistent with the previously suggested role for RecJ, a 5′→3′ DNA exonuclease, which may resect the 5′ overhang on the bottom strand resulting from the staggered double-strand break made by the group II intron RNP [12]. A surprising finding, however, was that bottom-strand DNA synthesis in extracts requires not only the group II intron RT, but is also strongly inhibited in extracts from DnaG primase mutants and moderately inhibited in extracts from DinB and PolB DNA repair polymerase mutants. The DnaG mutant extracts showed strongly decreased synthesis of even short cDNAs (Figure 4C), suggesting that DnaG may be needed for initiation of TPRT, possibly by functioning in conjunction with DNA repair polymerases to copy the 5′-top-strand DNA overhang before the group II intron RT is engaged to copy the reverse-spliced intron RNA. As LtrA can by itself efficiently initiate TPRT directly from the bottom-strand cleavage site in *in vitro* reactions with purified RNPs [16], [75], we speculate that host proteins, such as Ssb or RecA, may block initiation by the foreign group II intron RT in extracts, whereas host DNA repair enzymes have mechanisms for overcoming such blocks. Either or both mechanisms for initiation of TPRT could be employed *in vivo*.

We also identified a number of host proteins in which mutations inhibit retrohoming *in vivo*, but not *in vitro*. A number of these proteins act on chromosomal DNA or in transcription (*e.g.*, GyrB, Hns, RpoH, SbcC, Tus) and could impact group II intron retrohoming *in vivo* by affecting chromosome structure, DNA replication, or target site accessibility. Also affecting retrohoming *in vivo* but not in our *in vitro* assay is MnmE, which functions in tRNA modification and may affect the activity or intracellular levels of group II intron RNPs (see also [22], [30]). The failure to observe a decrease in retrohoming activity in some mutant extracts could also be due to replacement of the required activity by other host enzymes.

In addition to mutants that decrease retrohoming efficiency, our transposon-library screen also identified host genes whose disruption leads to increased retrohoming efficiencies. Although we hoped such mutants would identify a variety of host defense factors that function in different ways, all five transposon-insertions that increased retrohoming efficiency in our screen mapped to three closely linked genes associated with a cryptic prophage: *rnlA*, which encodes RNase LS; *yfjK*, which encodes a DExH/D-box helicase; and *yfjL*, which encodes a protein of unknown function. The identity of these genes and the finding that their disruption also leads to elevated GFP expression from a control reporter construct that lacks the Ll.LtrB intron suggest that they suppress retrohoming by degrading group II intron RNAs. Previous studies showed that mutations in RNase E, an essential protein, increase retrohoming frequencies by inhibiting group II intron RNA degradation [12], [22]. Together, these findings indicate that cellular RNases function as a major host defense mechanism for suppressing retrohoming. Additionally, our findings identify a new putative RNA degradation pathway in *E. coli* K12 that may have been acquired from another bacteria via insertion of a temperate phage and may constitute a second degradosome. The suppression of group II intron mobility by intron RNA degradation in bacteria may be analogous to the suppression of mobility of LINE-1 elements by sequestration into stress granules in human cells [76], [77].

Given our findings for Ll.LtrB retrohoming, we anticipate that replication restart proteins may also function in alternate group II intron retromobility pathways in which a nascent strand at a DNA replication fork rather than a cleaved DNA strand is used to prime reverse transcription of the intron RNA [78]–[80]. In these pathways, which are used by group II introns whose IEPs lack DNA endonuclease activity, reverse splicing is thought to result in the insertion of a group II intron RNP into a DNA target site ahead of a replication fork, with the RT positioned to use either a nascent leading or lagging strand as a primer for reverse transcription, depending upon the strand into which the intron inserted [80]. The stalling of the replication fork when it encounters the inserted group II intron RNP may lead first to dissociation of the replisome, enabling the group II intron RT to access the nascent DNA strand for the priming of cDNA synthesis, and then contribute to its re-recruitment via replication restart proteins for second-strand synthesis and continuation of host DNA replication. We note that yeast mtDNA group II introns primarily use a recombination mechanism rather than replication restart for cDNA integration (see Introduction), and the extent to which the replication restart, DNA recombination, or other pathways are used for the retromobility of different group II introns in different bacteria remains to be elucidated.

Key features of the Ll.LtrB intron retrohoming mechanism delineated here may be relevant to the propagation of LINES and other non-LTR-retrotransposons in eukaryotic nuclear genomes. Non-LTR-retrotransposons, which are thought to be evolutionary descendants of mobile group II introns, encode closely related RTs and use an analogous TPRT mechanism for cDNA synthesis [1], [7]. Like mobile group II introns, most non-LTR-retrotransposons do not encode RNase H and presumably rely on a cellular enzyme to degrade the RNA template strand after cDNA synthesis [7]. The mechanism used for second-strand DNA synthesis by non-LTR-retrotransposons is unknown, but given that non-LTR-retrotransposons carry out reverse transcription in the nucleus could well involve the use of a host DNA polymerase and replication restart proteins as found here for group II introns. The use of replication restart for second-strand synthesis by LINE-1 elements is consistent with their ability to retrotranspose in non-dividing cells [81]. In contrast to non-LTR-retroelements, retroviruses and LTR-containing retrotransposons carry out reverse transcription in the cytosol and rely on RTs that have acquired an RNase H domain and efficient DNA-dependent DNA polymerase activity to synthesize a pre-integration complex containing dsDNA that must then enter the nucleus for integration into the genome [7]. These evolutionary advances, which enable LTR-containing retroelements to carry out major steps of their replication pathway in the cytosol, may contribute to their greater propensity to be transferred horizontally between species and evolve into infectious viruses.

Finally, our results have implications for replication restart pathways. In *E. coli*, replication restart occurs on stalled or collapsed DNA replication forks and is thus dependent upon on-going DNA synthesis. Surprisingly, our extract assays indicate that replication restart components can synthesize a complete second-strand DNA without on-going DNA replication. These findings resemble recent results for bacteriophage Mu where PriA was found to be required for filling in 5-bp gaps at each end of the Mu insertion in the absence of DNA replication [82]. Thus, replication restart proteins may play a more general role both in the repair of DNA damage and propagation of mobile elements than was thought previously, including as an integral part of the group II intron retrohoming mechanism.

## Materials and Methods

### *E. coli* strains and growth conditions

*E. coli* HMS174(DE3) (Novagen) was used for the transposon library screen and retrohoming assays and DH5α was used for cloning. The construction of the *mariner* transposon library in HMS174(DE3) was described previously [83]. *E. coli* Keio deletions and their parental wild-type strain BW25113 were obtained from the National BioResource Project (National Institute of Genetics, Japan). Wild-type SS996 and mutant strains in this genetic background were obtained from Dr. Steven Sandler (University of Massachusetts) [84]. A complete listing of strains and genotypes is given in Table S7.

Cells were grown in Luria-Bertani (LB), Mueller-Hinton (MH) [85], or 2xYT medium [86], as specified for individual experiments. Antibiotics were added at the following concentrations: ampicillin, 100 µg/ml; chloramphenicol, 25 µg/ml; kanamycin, 40 µg/ml; rifampicin, 50 µg/ml; trimethoprim (Tp), 10 µg/ml. Thymine was added at 2 µg/ml.

### Recombinant plasmids

The intron-donor plasmids pALG2 (Figure S2A; [48]) and pALG3 (Figure 2A) have the vector backbone of pACYC184 with a *cam^R^* marker and use a T7lac promoter to express an *ltrB*/GFP fusion cassette, followed by the LtrA ORF. The *ltrB*/GFP fusion cassette consists of the Shine-Dalgarno sequence and the first eight codons of the phage T7 Φ10 gene linked in-frame to a segment of the *L. lactis ltrB* gene [58-bp exon 1 (E1), 915-bp Ll.LtrB-ΔORF intron, and 38-bp exon 2 (E2)], with E2 linked to codons 2 to 238 of the GFP ORF. The GFP ORF is derived from pGFPuv (Clontech), which has the amino acid substitutions F64L and S65T to improve performance in FACS assays [87]. The LtrA ORF is cloned downstream of the *ltrB*/GFP fusion cassette and has its own Shine-Dalgarno sequence. pALG3 additionally contains a trimethoprim-resistance retrotransposition-activated marker (Tp^R^-RAM [24]) inserted at the MluI site in DIV of the Ll.LtrB-ΔORF intron, enabling selection for retrohoming events.

The intron-recipient plasmid pBRR3-ltrB (Figure 2A) is a derivative of pBR322 with an *amp^R^* marker [26]. It contains a 45-bp wild-type *ltrB* target site cloned upstream of a promoterless *tet^R^* gene, enabling detection of mobility events either by using the Tp^R^-RAM marker, as in the present work, or by integration of an intron carrying a phage T7 promoter for reporter gene activation [26].

The control plasmid pALE is a derivative of pALG2 that lacks the Ll.LtrB-ΔORF intron and has the ligated *ltrB* exon sequences linked directly to GFP (Figure S2B; [48]).

pBL1Cap is a broad host range intron-donor plasmid that uses an *m*-toluic acid-inducible promoter to express the Ll.LtrB-ΔORF intron and flanking exons. It was derived from the broad host range intron-donor plasmid pBL1 [28] by replacing the *tet^R^* marker with a *cam^R^* marker (1.5-kb NheI/PshAI fragment of pACD3 blunt ended and cloned in place of *tet^R^* between the FspI sites of the pBL1).

pBL1-rhlE is a derivative of pBL1Cap that expresses an Ll.LtrB-ΔORF intron that was retargeted to insert into a site in the antisense strand of the *rhlE* gene [21].

### Transposon-library screen and Tp^R^-RAM selection assay

The intron-donor plasmid pALG3 and recipient plasmid pBRR3-ltrB were co-transformed into HMS174(DE3) containing random *mariner*-transposon insertions [83], and the cells were plated on LB medium containing ampicillin and chloramphenicol to select for the markers on the plasmids. Individual colonies were resuspended in 500 µl of LB medium containing the same antibiotics in 96-deep-well plates and incubated overnight at 37°C. A portion (20 µl) of the overnight culture was transferred into 500 µl of fresh LB medium containing ampicillin and chloramphenicol in new 96-deep-well plates, incubated with shaking at 37°C for 2 h, and then induced with 0.5 mM IPTG for 3 h at 30°C. After induction, 10^5^ cells from each sample were transferred to another set of 96-deep-well plates containing 600 µl of MH medium or MH medium with thymine and trimethoprim. The plates were then incubated overnight at 30°C, and the O.D._595_ of individual wells was read by a DTX880 multimode plate reader (Beckman Coulter). DNA was isolated by using a Bacterial Genomic DNA Prep kit (Qiagen) and transposon-insertion sites were mapped by Thermal-Asymmetric-Interlaced (TAIL) PCR [25]. Southern hybridizations to confirm that each mutant contained a single transposon insertion at the identified site were as described [83].

### FACS analysis of GFP expression

*E. coli* strains carrying plasmids with *ltrB*/GFP fusions cassettes were grown to log phase (O.D._595_ = 0.2–0.4) and induced for 3 h at 30°C with 0.1 mM IPTG (pALG2 or pALE) or 0.5 mM IPTG (pALG3). Wild-type and mutant SS996 (P*sulA*-GFP) strains were grown at 30°C to O.D._595_ = 0.2–0.3 and induced with 4 mM *m*-toluic acid for 1 h at 37°C. Cells were collected by centrifugation, resuspended in phosphate-buffered saline (140 mM NaCl, 2.7 mM KCl, 9 mM Na_2_HPO_4_, 1.6 mM KH_2_PO_4_, pH 7.4), and analyzed by using a FACS Caliber (Becton Dickinson), with filter FL1 set at 530±30 nm. FACS data were analyzed with the CELLQuest Pro program (Becton Dickinson).

### Taqman qPCR assay of retrohoming

Retrohoming frequencies in Keio deletion and temperature-sensitive mutant strains were determined by using a retargeted Ll.LtrB-ΔORF intron (rhlE481a) that inserts at a site in the chromosomal *rhlE* gene [21] and quantifying the 5′- and 3′-integration junctions relative to the number of *rhlE* genes by Taqman qPCR. For this assay, *E. coli* strains were transformed with pBL1-rhlE and grown overnight at 37°C in LB medium containing chloramphenicol. The overnight culture was subcultured into fresh medium and incubated at 37°C until O.D._595_ = 0.2–0.3. Then, triplicate 3-ml cultures were induced with 4 mM *m*-toluic acid for 1 h at 37°C. The *priA* Keio deletion strain was grown and induced at 30°C to avoid the accumulation of suppressor mutations. Five temperature-sensitive mutants (*dnaE^ts^*, *gyrB^ts^*, *ligA^ts^*, *rpoH^ts^*, and *ssb^ts^*) that could not be grown at 37°C were grown at 30°C and then shifted to 37°C for 1 h followed by a 1-h induction with *m*-toluic acid. After induction, cells were lysed immediately and DNA was extracted by using a DNeasy Blood and Tissue Kit (Qiagen).

Taqman qPCR was carried out on 10 ng of total DNA in 384-well plates using a Universal qPCR Master Mix kit (Applied Biosystems), with the following primer/probe sets: (i) 5′-integration junction. P5-forward 5′-GGTGCAAACCAGTCACAGTAATG; reverse 5′-GTCAGCTTCATCGAGGACGAG; Taqman probe 5′-CAAGGCGGTACCTCC; (ii) 3′-integration junction. P3-forward 5′-ATAAAGCCCATGTCGAGCATG; reverse 5′-TGTAAGATAACACAGAAAACAGCCAA; Taqman probe 5′-TGCGCCCAGATAGGGTGTTAAGTCAAGTAGT; (iii) *rhlE* gene. rhlE-forward 5′-CAGCAACGTCCCGGGG; reverse 5′-ACGCAGTTTCATCATCTGCG; Taqman probe 5′-CCACCAGCACATCAACGCCGC. Taqman qPCR primers and probes with a 5′ FAM (6-carboxyfluorescein) label and 3′ MGB (dihydrocyclopyrroloindole tripeptide major groove binder) quencher were obtained from Applied Biosystems. Standard curves were generated by serial ten-fold dilutions of a TOPO-2.1 vector carrying a cloned DNA fragment containing the Ll.LtrB-ΔORF intron integrated within the *rhlE* gene. All primer/probe sets had >90% amplification efficiency over the concentration range of the standard curve. Retrohoming frequencies were calculated as the numbers of 5′- and 3′-integration junctions relative to the number of *rhlE* genes after subtraction of background signal without *m*-toluic acid induction and are the mean ± standard error of the mean (S.E.M.) for triplicate 1-h inductions.

### *E. coli* extract assays

*E. coli* S12 extracts were prepared by a modification of procedures used to prepare extracts for *in vitro* transcription and translation [61], [62]. Cells were grown at 37°C in 200 ml of 2xYT medium at 200 reciprocations per min to O.D._595_ = 0.8–1.0, harvested by centrifugation, and washed three times with 20 ml of buffer A [10 mM Tris-acetate, pH 8.2, 14 mM magnesium acetate, 60 mM potassium glutamate, 1 mM dithiothreitol (DTT), and 0.05% (v/v) 2-mercaptoethanol (2-ME)]. The five temperature-sensitive strains (*dnaE^ts^*, *gyrB^ts^*, *ligA^ts^*, *rpoH^ts^*, and *ssb^ts^*) that could not be grown at 37°C were instead grown to O.D._595_ = 0.4–0.6 at 30°C, then shifted to 37°C and incubated for an additional 2 h. The *priA* deletion strain was grown at 30°C. After growth, the cells were resuspended in 1.27 ml of buffer B (buffer A without 2-ME) per g wet weight and disrupted by using a BeadBeater [3.24 g of 0.1-mm glass beads (BioSpec Products) per g wet weight; 3 cycles of 1 min at 4°C followed by 1-min incubation on ice]. The crude lysate was centrifuged twice (12,000× g for 10 min at 4°C), and the supernatant was removed and pre-incubated at 37°C for 30 min to complete endogenous reactions and release required components. Extracts prepared without this pre-incubation step showed no detectable top-strand DNA synthesis in retrohoming assays. The extracts were divided into 50-µl aliquots and stored at −80°C. Cell extracts from different strains were checked by Coomassie blue staining of SDS-polyacrylamide gels to confirm that they contained equal amounts of total protein.

The DNA substrates used for extract assays consisted of the top-strand oligonucleotide (5′- GCAACCCACGTCGATCGTGAACACATCCATAACCATATCATTTTTAATTCTACGAATCTTTATACTGGCAAAC) and the bottom-strand oligonucleotide (5′- GTTTGCCAGTATAAAGATTCGTAGAATTAAAAATGATATGGTTATGGATGTGTTCACGATCGACGTGGGTTGC). The strands were annealed by mixing 1 µM of each strand in RNase-free water, heating to 100°C for 5 min, and slowly cooling to room temperature. DNA oligonucleotides were 5′-labeled with ^32^P using T4 polynucleotide kinase (New England Biolabs), according to the manufacturer's protocol.

Assays were carried out in 20 µl of reaction mixture containing 50 nM DNA substrate, 3 µl of *in vitro* reconstituted Ll.LtrB RNPs (5–10 µg based on O.D._260_; RNPs prepared as described in ref. [13]), 6 µl of S12 extract, 20 µM carrier DNA oligonucleotide (5′-GTGATGTCTGAAAAGAACGGGAAG) as protection against DNase activity, 56.4 mM Tris-acetate buffer, pH 7.5, 100 mM potassium acetate, 35.9 mM ammonium acetate, 24 mM magnesium acetate, 1.5 mM ATP, 1 mM each of dATP, dCTP, dGTP, and dTTP (collectively denoted dNTPs), 500 µM of CTP, GTP, and UTP, 5 mM phosphoenolpyruvate, 50 µg/ml pyruvate kinase, 2 units/µl RNaseOUT (Invitrogen), and 1% (v/v) protease inhibitor cocktail [made by dissolving a mini EDTA-free tablet (Roche) in 1 ml of RNase-free water]. For time-course experiments, the reactions were scaled up to 100 µl, and a 20-µl portion was withdrawn at each time point. Reactions were initiated by adding labeled DNA substrate, incubated at 37°C for times specified for individual experiments, and terminated by extraction with phenol-chloroform-isoamyl alcohol (phenol-CIA; 25∶24∶1 by volume). After digestion with proteinase K (2 µg/µl, 30 min at 37°C) and re-extraction with phenol-CIA, nucleic acids were ethanol precipitated and dissolved in 20 µl of nuclease-free water. For RNase treatment, 0.4 units RNase H (Invitrogen) and 0.1 µg RNase A (Roche) were added, and the sample was incubated for 30 min at 37°C before the ethanol-precipitation step. Samples were analyzed in a denaturing 6% polyacrylamide gel, which was dried and scanned with a PhosphorImager.

For primer-extension analysis, DNA products were separated in a 1% low-melting point agarose gel (Fermentas). A gel slice containing DNAs of 0.85–1.2 kb was heated to 70°C for 10 min and then digested with agarase (Fermentas) for 30 min at 42°C. The released DNAs were ethanol precipitated in the presence of glycogen carrier (Fermentas), washed with 70% ethanol, and dissolved in 10 µl of RNase-free water. For primer extension, a 1-µl portion of the DNA was incubated with 10 µM labeled primer in a 10-µl PCR reaction (95°C, 5 min; then 10–20 cycles of 95°C, 15 sec, 58°C, 30 sec, slow ramp to 72°C, 60 sec; 72°C, 4 min) in an ABI 9700 PCR apparatus. Primers were FB, 5′-TCGATCGTGAACACATCCATAAC; 5T, 5′-TGCTCTGTTCCCGTATCAGCT; and FT, 5′-GTTTGCCAGTATAAAGATTCGTAGAA. Samples were analyzed in a denaturing 6% polyacrylamide gel, which was dried and scanned with a PhosphorImager.

## Supporting Information

Figure S1 Transposon library screen. After transformation of intron-donor plasmid pALG3 and recipient plasmid pBRR3-ltrB (Figure 2A) into *E. coli* HMS174(DE3) containing randomly inserted *mariner* transposons, colonies were picked and grown to mid-log phase in 96-well plates and induced with 0.5 mM IPTG for 3 h at 30°C. A portion of each well (10^5^ cells based on O.D._595_) was then transferred to 96-well plates with MH medium or MH medium plus trimethoprim and thymine, and grown overnight at 30°C with shaking. The growth rate of each mutant was quantified by determining O.D._595_ with a plate reader and correcting for background by subtracting O.D._595_ of a blank containing MH medium alone. The ratio of O.D._595_ under the selective conditions to that under the non-selective conditions provides a measure of retrohoming efficiency. Control wells on each 96-well plate were: A1, MH medium only, used as a blank for the plate reader (CC); A2, assay control (AC), wild-type HMS174(DE3) without donor or recipient plasmids, Tp^S^/GFP^−^ phenotype; A3, negative control (NC), wild-type HMS174(DE3) containing pALG2 (no Tp^R^-RAM marker) and pBRR3-ltrB, Tp^S^/GFP^+^ phenotype; and A4, positive control (PC), wild-type HMS174(DE3) containing pALG3 and pBRR3-ltrB, Tp^R^/GFP^+^ phenotype. Candidate mutants were picked to new 96-well plates and re-screened by selection with trimethoprim to confirm the Tp^S^ phenotype and FACS assay to quantify GFP expression. After screening 9,200 colonies by two rounds of 96-well plate assays and eliminating false positives that were Tp^S^ due to retention of mariner transposon expression plasmid pSC189 [83], which is Kan^R^+Amp^R^ and interferes with transformation of the Amp^R^ recipient plasmid pBRR3-ltrB (172/9165 = 0.19%), we identified 61 transposon-insertion mutants that reproducibly had a >4-fold decrease in retrohoming efficiency compared to the positive control and were GFP^+^ by FACS assay. Eight mutants had decreased retrohoming efficiency and were Tp^S^ GFP^−^ (Figure S3 and Table S3), and five mutants had increased retrohoming efficiency and increased GFP expression (Tp^R^/GFP^++^) (Figure S4 and Table S5). Transposon insertion mutants are named according to a plate number (01-100), followed by row (A–H), and column number (01-12).
(TIF)

Figure S2 Plasmid pALG2 used to screen for mutants defective in RNA splicing and the control plasmid pALE. (A) pALG2 is a Cam^R^ pACYC184-based intron donor plasmid that uses a T7lac promoter to express an *ltrB*/GFP fusion cassette followed by the LtrA ORF. The *ltrB*/GFP cassette contains the Ll.LtrB-ΔORF intron and flanking 5′- and 3′-exons (E1 and E2, respectively), with the 3′ exon linked in-frame to the GFP. (B) The control plasmid pALE is identical to pALG2 but lacks the Ll.LtrB-ΔORF intron leaving the ligated *ltrB* exon sequence (E1–E2) fused directly to the GFP ORF.
(TIF)

Figure S3 FACS analysis of GFP expression from the control plasmid pALE lacking the Ll.LtrB intron in Tp^S^/GFP^−^ mutants. Cells were grown to mid-log phase (O.D._595_ = 0.2–0.4) and induced with 0.1 mM IPTG for 3 h at 30°C. The plots show cell counts as a function of fluorescence intensity. Black and red, GFP fluorescence from wild-type HMS174(DE3) containing pALE without and with IPTG induction, respectively; green, GFP fluorescence from pALE after IPTG induction in transposon-insertion mutants in the indicated genes (strain numbers indicated in parentheses). All of the mutants show decreased levels of GFP fluorescence relative to the wild-type strain from the control plasmid pALE after IPTG induction, indicating that decreased GFP expression is not due to a defect in RNA splicing.
(TIF)

Figure S4 FACS analysis of GFP expression from pALG2 and pALE in mutant strains with increased retrohoming efficiencies. (A) Map of the *E. coli* chromosome region encoding *yfjK*, *yfjL*, and *rnlA*, which are sites of transposon insertions that result in increased retrohoming efficiency. Arrows indicates the direction of transcription. Transposon-insertion sites, all of which are in the (-) strand, are shown below. (B) and (C) FACS assays of GFP expression from pALG2 and pALE, respectively. Cells were grown to mid-log phase (O.D._595_ = 0.2–0.4) and induced or not induced with 0.1 mM IPTG for 3 h at 30°C, as indicated below. The plots show cell counts as a function of fluorescence intensity. (B) Black, basal fluorescence in wild-type HMS174(DE3) without IPTG induction; red, GFP fluorescence from pALG2 in wild-type HMS174(DE3) after IPTG induction; green, GFP fluorescence from pALG2 after IPTG induction in transposon-insertion mutants in the indicated genes (strain numbers indicated in parentheses). (C) FACS assays of GFP expression from pALE without IPTG induction. Black, fluorescence from wild-type HMS174(DE3); red, GFP fluorescence from pALE in wild-type HMS174(DE3); green, GFP fluorescence from pALE in transposon-insertion mutants in the indicated genes (strain numbers indicated in parentheses). All of the mutants show increased levels of GFP fluorescence relative to the wild-type strain from both pALG3 and the control plasmid pALE irrespective of the presence or absence of the Ll.LtrB intron.
(TIF)

Figure S5 Decreased retrohoming frequencies in replication restart mutants are not due to the SOS response. *E. coli* SS996 P*sulA*-GFP strains with mutations in genes encoding replication restart proteins were grown in LB medium at 30°C until O.D._595_ = 0.2–0.4, then shifted to 37°C and induced with 4 mM *m*-toluic acid for 1 h. Retrohoming frequencies were determined by Taqman qPCR assay of retrohoming into a chromosomal target site in the *rhlE* relative to the number of available *rhlE* target sites, and SOS induction in the same cultures was assessed by the difference (Δ) in the percentage of cells showing GFP fluorescence in a FACS assay before (pre) and after (post) the shift to 37°C. The error bars indicate the S.E.M. for three separate *m*-toluic acid-induced cultures.
(TIF)

Figure S6 Stability of single- and double-stranded DNA in *E. coli* extracts under the conditions of the biochemical assays. 5′ ^32^P-labeled double-stranded (ds) and single-stranded (ss) DNAs were incubated without (“None”) or with S12 extract from the indicated *E. coli* strains under the conditions used for biochemical assays. The dsDNA is similar in length and sequence to the double-stranded retrohoming product in biochemical assays, and it was generated by PCR of the Ll.LtrB-ΔORF intron and flanking exons using pBL1Cap as template with primers F (5′-TCGTGAACACATCCATAAC) and R (5′-GCGATGCTGTCGGAATGGAC). The ssDNA corresponds to the product of bottom-strand cDNA synthesis in the biochemical assays, and it was generated by primer extension of the control dsDNA using primer R. Both the dsDNA and ssDNA were gel-purified and 5′ ^32^P-labeled using T4 polynucleotide kinase (New England Biolabs). Similar controls indicated that the DnaT deletion mutant has elevated phosphatase activity that removes the 5′-end label (not shown).
(TIF)

Figure S7 Assays of top- and bottom-strand DNA synthesis in extracts from *E. coli* mutant strains that did not show substantially decreased top- or bottom-strand synthesis. DNA substrates labeled at the 5′ end of either the top (T) or bottom (B) strand were incubated with group II intron RNPs for 15 min at 37°C in reaction medium containing extracts from: (A) Keio deletion mutants and their parental wild-type strain BW25113. (B) Temperature-sensitive mutants and their parental wild-type strains. After phenol-CIA extraction and proteinase K digestion, samples were split into halves that were incubated without or with RNases A+H for 30 min at 37°C. The products were analyzed in a denaturing 6% polyacrylamide gel, which was dried and scanned with a PhosphorImager. Extracts were confirmed to contain equal amounts of protein by SDS-polyacrylamide gels stained with Coomassie blue (not shown). The amount of radiolabel in the top- and bottom-strand products was normalized for the amount of substrate (S) in each lane and expressed as a percent of that in the parental wild-type strain, with results summarized in Table 2 and Table S6. At least two assays were done for each mutant and were reproducible to within <30%.
(TIF)

Table S1 Genetic assay of retrohoming efficiencies of all strains identified as Tp^S^/GFP^+^ in the transposon-library screen.
(DOCX)

Table S2 Taqman qPCR assays of retrohoming in other *E. coli* Keio deletion mutants analyzed in this work.
(DOCX)

Table S3 *E. coli* transposon-insertion mutants identified as Tp^S^/GFP^−^ in the transposon-library screen.
(DOCX)

Table S4 *E. coli* transposon mutants identified at GFP^−^ in an *in vivo* assay in which splicing of the Ll.LtrB intron is linked to GFP expression.
(DOCX)

Table S5 *E. coli* mutants identified as having increased retrohoming efficiencies in the transposon-library screen.
(DOCX)

Table S6 *E. coli* extract assays of retrohoming in wild-type and additional Keio deletion mutant strains.
(DOCX)

Table S7 *E. coli* strains used in this work.
(DOCX)
