# Supplementary figures and images for: Genetic and Biochemical Assays Reveal a Key Role for Replication Restart Proteins in Group II Intron Retrohoming
Source: PLoS Genet. 2013 Apr 25;9(4):e1003469. doi: 10.1371/journal.pgen.1003469 (PMC3636086; doi:10.1371/journal.pgen.1003469)

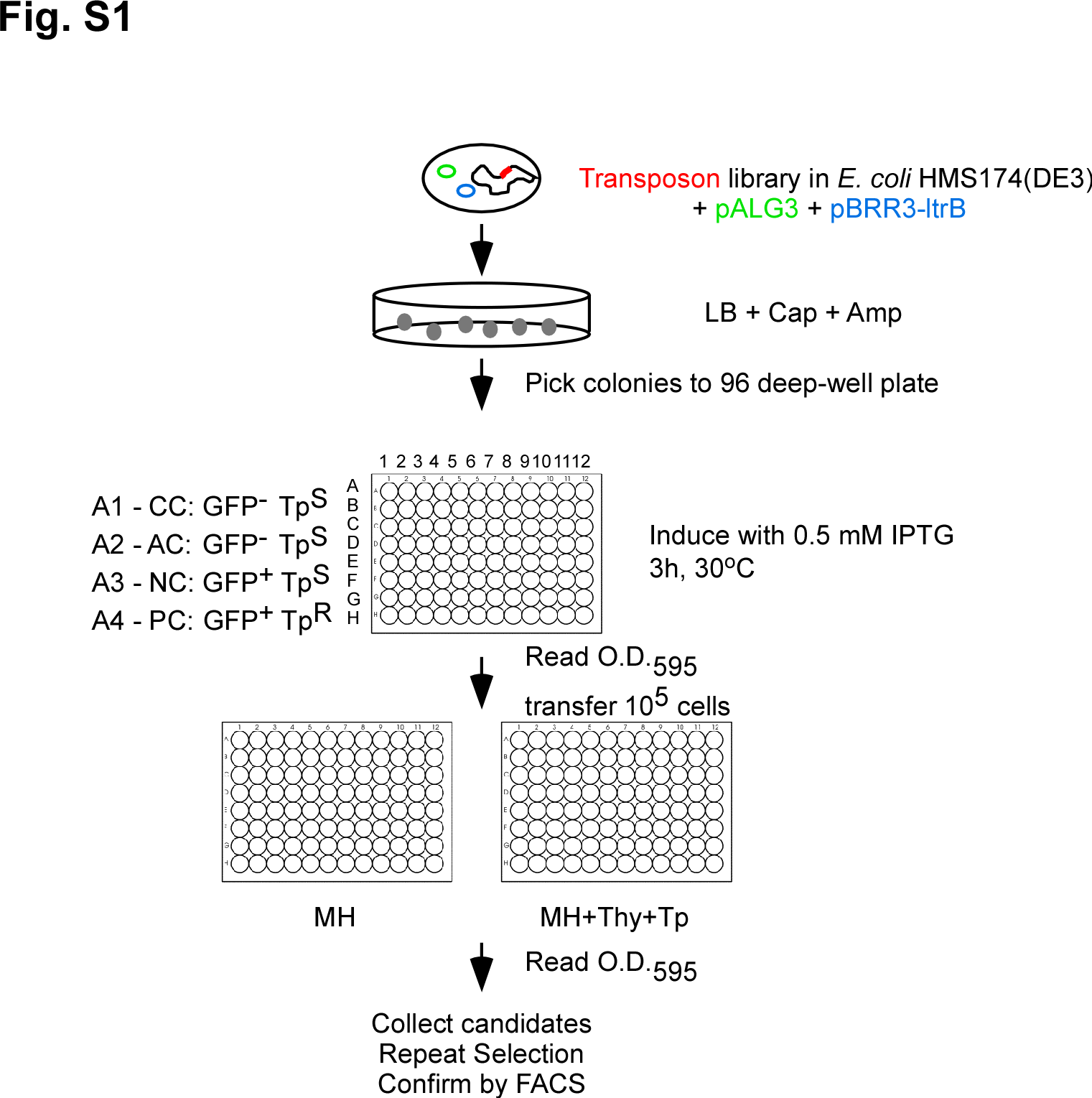

Supplement: Figure S1 — Transposon library screen. After transformation of intron-donor plasmid pALG3 and recipient plasmid pBRR3-ltrB (Figure 2A) into E. coli HMS174(DE3) containing randomly inserted mariner transposons, colonies were picked and grown to mid-log phase in 96-well plates and induced with 0.5 mM IPTG for 3 h at 30°C. A portion of each well (105 cells based on O.D.595) was then transferred to 96-well plates with MH medium or MH medium plus trimethoprim and thymine, and grown overnight at 30°C with shaking. The growth rate of each mutant was quantified by determining O.D.595 with a plate reader and correcting for background by subtracting O.D.595 of a blank containing MH medium alone. The ratio of O.D.595 under the selective conditions to that under the non-selective conditions provides a measure of retrohoming efficiency. Control wells on each 96-well plate were: A1, MH medium only, used as a blank for the plate reader (CC); A2, assay control (AC), wild-type HMS174(DE3) without donor or recipient plasmids, TpS/GFP− phenotype; A3, negative control (NC), wild-type HMS174(DE3) containing pALG2 (no TpR-RAM marker) and pBRR3-ltrB, TpS/GFP+ phenotype; and A4, positive control (PC), wild-type HMS174(DE3) containing pALG3 and pBRR3-ltrB, TpR/GFP+ phenotype. Candidate mutants were picked to new 96-well plates and re-screened by selection with trimethoprim to confirm the TpS phenotype and FACS assay to quantify GFP expression. After screening 9,200 colonies by two rounds of 96-well plate assays and eliminating false positives that were TpS due to retention of mariner transposon expression plasmid pSC189 [83], which is KanR+AmpR and interferes with transformation of the AmpR recipient plasmid pBRR3-ltrB (172/9165 = 0.19%), we identified 61 transposon-insertion mutants that reproducibly had a >4-fold decrease in retrohoming efficiency compared to the positive control and were GFP+ by FACS assay. Eight mutants had decreased retrohoming efficiency and were TpS GFP− (Figure S3 and Table S3) [file pgen.1003469.s001.tif]

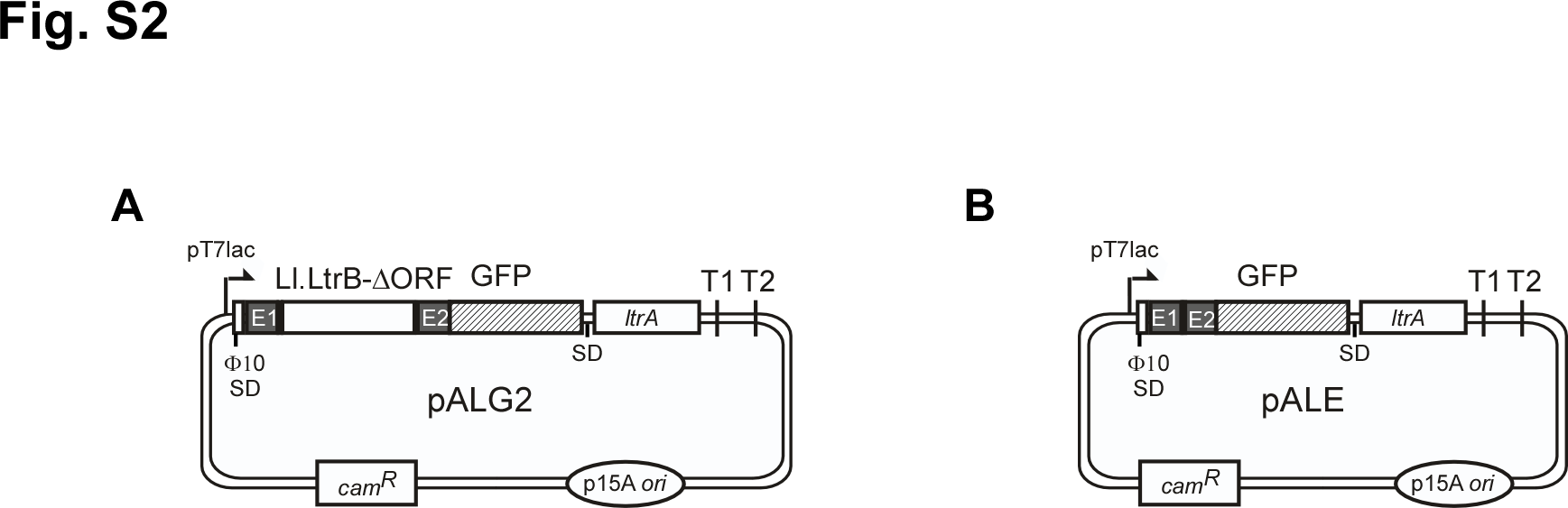

Supplement: Figure S2 — Plasmid pALG2 used to screen for mutants defective in RNA splicing and the control plasmid pALE. (A) pALG2 is a CamR pACYC184-based intron donor plasmid that uses a T7lac promoter to express an ltrB/GFP fusion cassette followed by the LtrA ORF. The ltrB/GFP cassette contains the Ll.LtrB-ΔORF intron and flanking 5′- and 3′-exons (E1 and E2, respectively), with the 3′ exon linked in-frame to the GFP. (B) The control plasmid pALE is identical to pALG2 but lacks the Ll.LtrB-ΔORF intron leaving the ligated ltrB exon sequence (E1–E2) fused directly to the GFP ORF. (TIF) [file pgen.1003469.s002.tif]

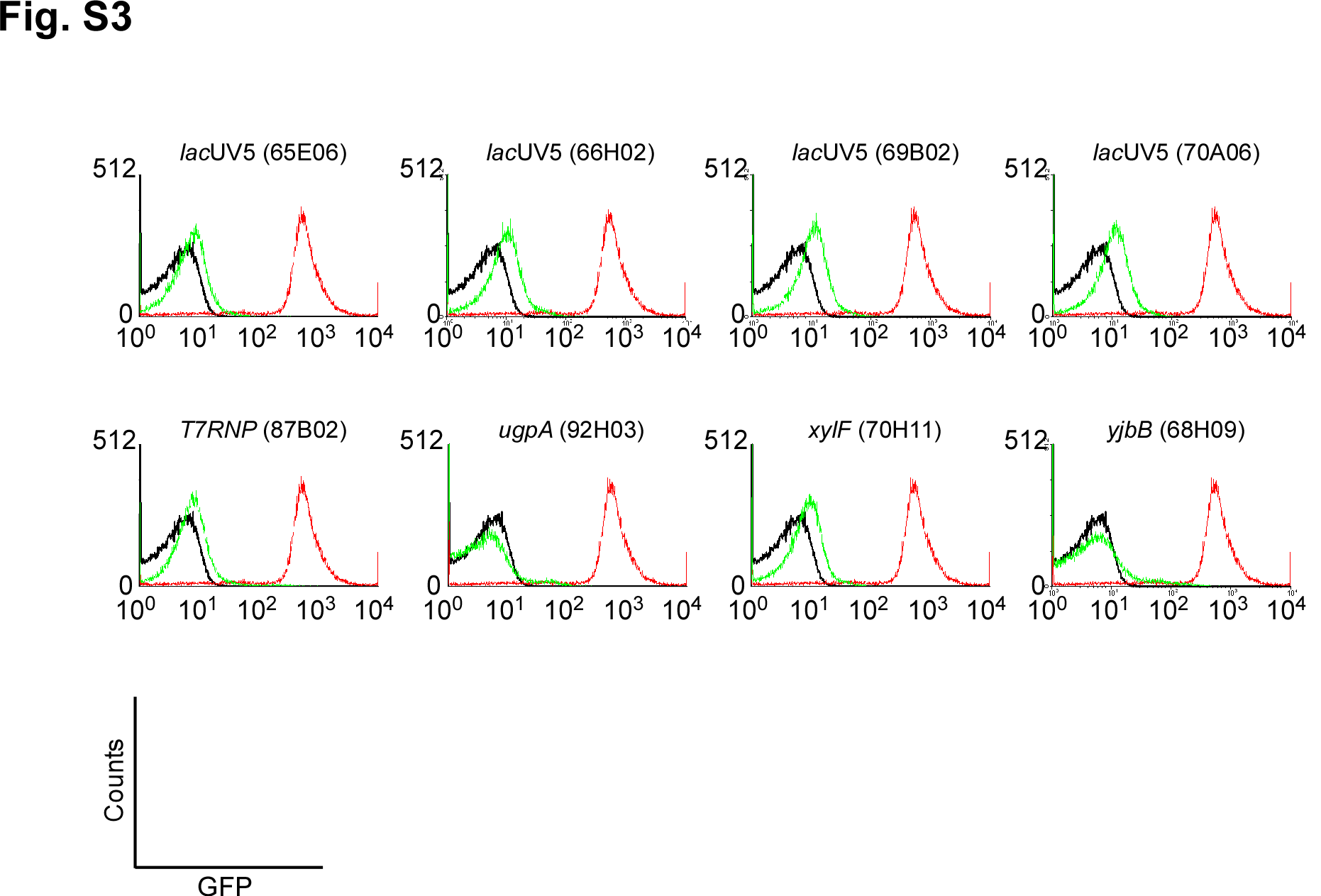

Supplement: Figure S3 — FACS analysis of GFP expression from the control plasmid pALE lacking the Ll.LtrB intron in TpS/GFP− mutants. Cells were grown to mid-log phase (O.D.595 = 0.2–0.4) and induced with 0.1 mM IPTG for 3 h at 30°C. The plots show cell counts as a function of fluorescence intensity. Black and red, GFP fluorescence from wild-type HMS174(DE3) containing pALE without and with IPTG induction, respectively; green, GFP fluorescence from pALE after IPTG induction in transposon-insertion mutants in the indicated genes (strain numbers indicated in parentheses). All of the mutants show decreased levels of GFP fluorescence relative to the wild-type strain from the control plasmid pALE after IPTG induction, indicating that decreased GFP expression is not due to a defect in RNA splicing. (TIF) [file pgen.1003469.s003.tif]

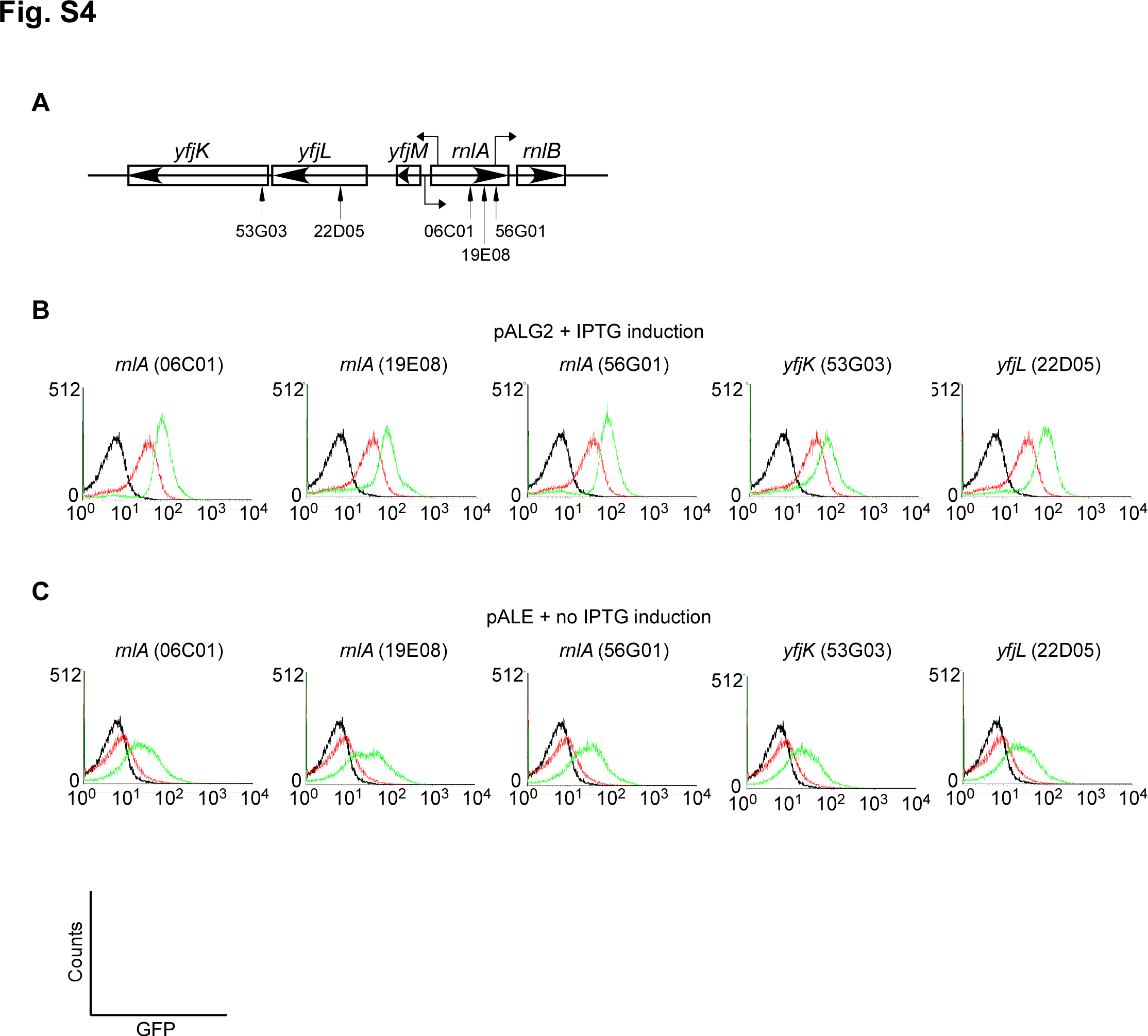

Supplement: Figure S4 — FACS analysis of GFP expression from pALG2 and pALE in mutant strains with increased retrohoming efficiencies. (A) Map of the E. coli chromosome region encoding yfjK, yfjL, and rnlA, which are sites of transposon insertions that result in increased retrohoming efficiency. Arrows indicates the direction of transcription. Transposon-insertion sites, all of which are in the (-) strand, are shown below. (B) and (C) FACS assays of GFP expression from pALG2 and pALE, respectively. Cells were grown to mid-log phase (O.D.595 = 0.2–0.4) and induced or not induced with 0.1 mM IPTG for 3 h at 30°C, as indicated below. The plots show cell counts as a function of fluorescence intensity. (B) Black, basal fluorescence in wild-type HMS174(DE3) without IPTG induction; red, GFP fluorescence from pALG2 in wild-type HMS174(DE3) after IPTG induction; green, GFP fluorescence from pALG2 after IPTG induction in transposon-insertion mutants in the indicated genes (strain numbers indicated in parentheses). (C) FACS assays of GFP expression from pALE without IPTG induction. Black, fluorescence from wild-type HMS174(DE3); red, GFP fluorescence from pALE in wild-type HMS174(DE3); green, GFP fluorescence from pALE in transposon-insertion mutants in the indicated genes (strain numbers indicated in parentheses). All of the mutants show increased levels of GFP fluorescence relative to the wild-type strain from both pALG3 and the control plasmid pALE irrespective of the presence or absence of the Ll.LtrB intron. (TIF) [file pgen.1003469.s004.tif]

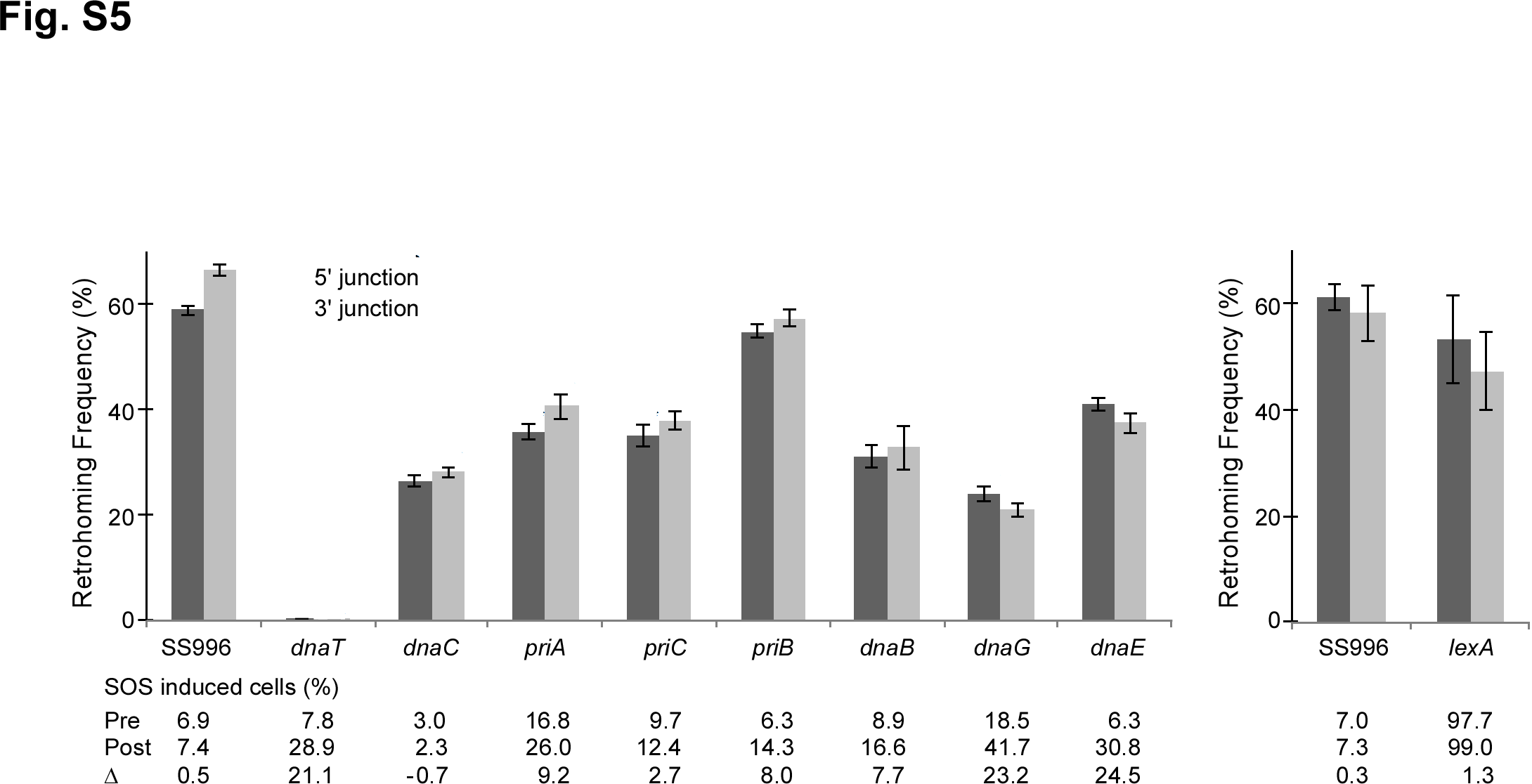

Supplement: Figure S5 — Decreased retrohoming frequencies in replication restart mutants are not due to the SOS response. E. coli SS996 PsulA-GFP strains with mutations in genes encoding replication restart proteins were grown in LB medium at 30°C until O.D.595 = 0.2–0.4, then shifted to 37°C and induced with 4 mM m-toluic acid for 1 h. Retrohoming frequencies were determined by Taqman qPCR assay of retrohoming into a chromosomal target site in the rhlE relative to the number of available rhlE target sites, and SOS induction in the same cultures was assessed by the difference (Δ) in the percentage of cells showing GFP fluorescence in a FACS assay before (pre) and after (post) the shift to 37°C. The error bars indicate the S.E.M. for three separate m-toluic acid-induced cultures. (TIF) [file pgen.1003469.s005.tif]

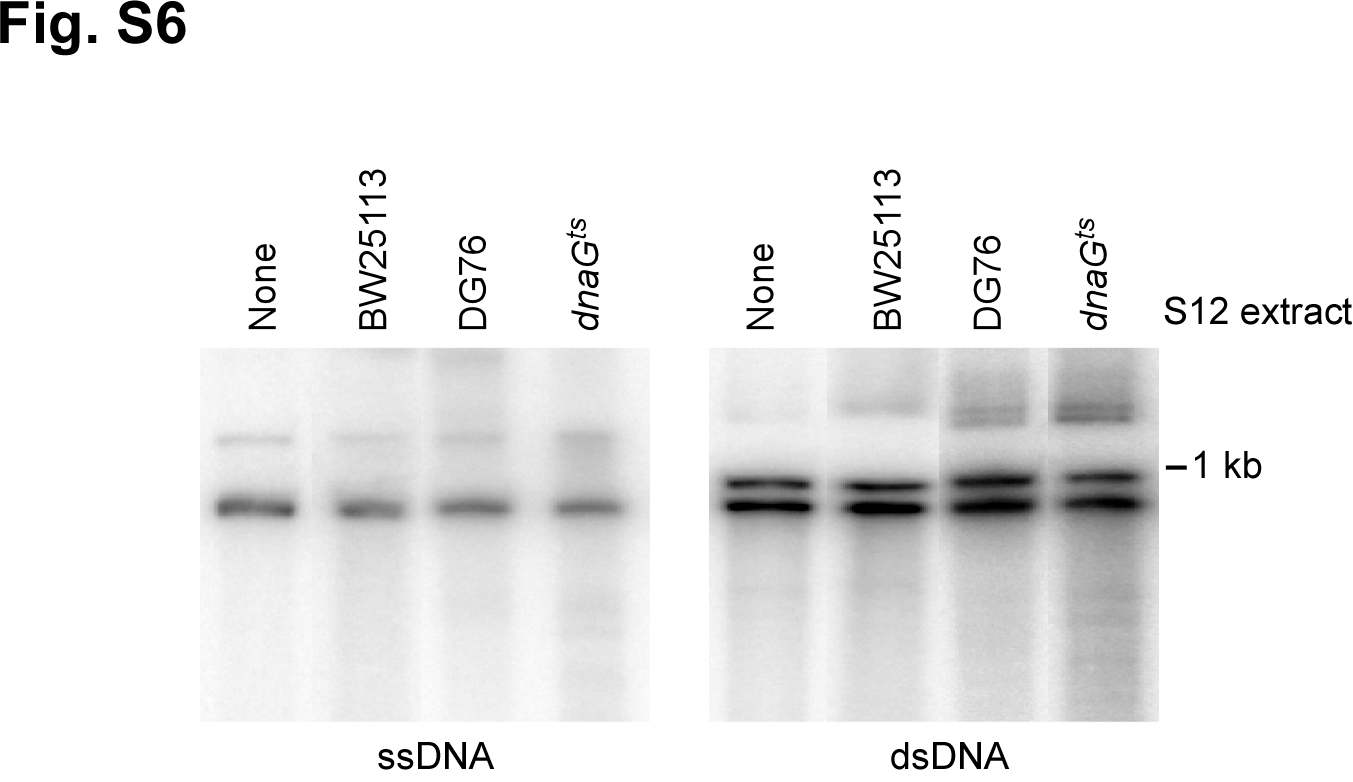

Supplement: Figure S6 — Stability of single- and double-stranded DNA in E. coli extracts under the conditions of the biochemical assays. 5′ 32P-labeled double-stranded (ds) and single-stranded (ss) DNAs were incubated without (“None”) or with S12 extract from the indicated E. coli strains under the conditions used for biochemical assays. The dsDNA is similar in length and sequence to the double-stranded retrohoming product in biochemical assays, and it was generated by PCR of the Ll.LtrB-ΔORF intron and flanking exons using pBL1Cap as template with primers F (5′-TCGTGAACACATCCATAAC) and R (5′-GCGATGCTGTCGGAATGGAC). The ssDNA corresponds to the product of bottom-strand cDNA synthesis in the biochemical assays, and it was generated by primer extension of the control dsDNA using primer R. Both the dsDNA and ssDNA were gel-purified and 5′ 32P-labeled using T4 polynucleotide kinase (New England Biolabs). Similar controls indicated that the DnaT deletion mutant has elevated phosphatase activity that removes the 5′-end label (not shown). (TIF) [file pgen.1003469.s006.tif]

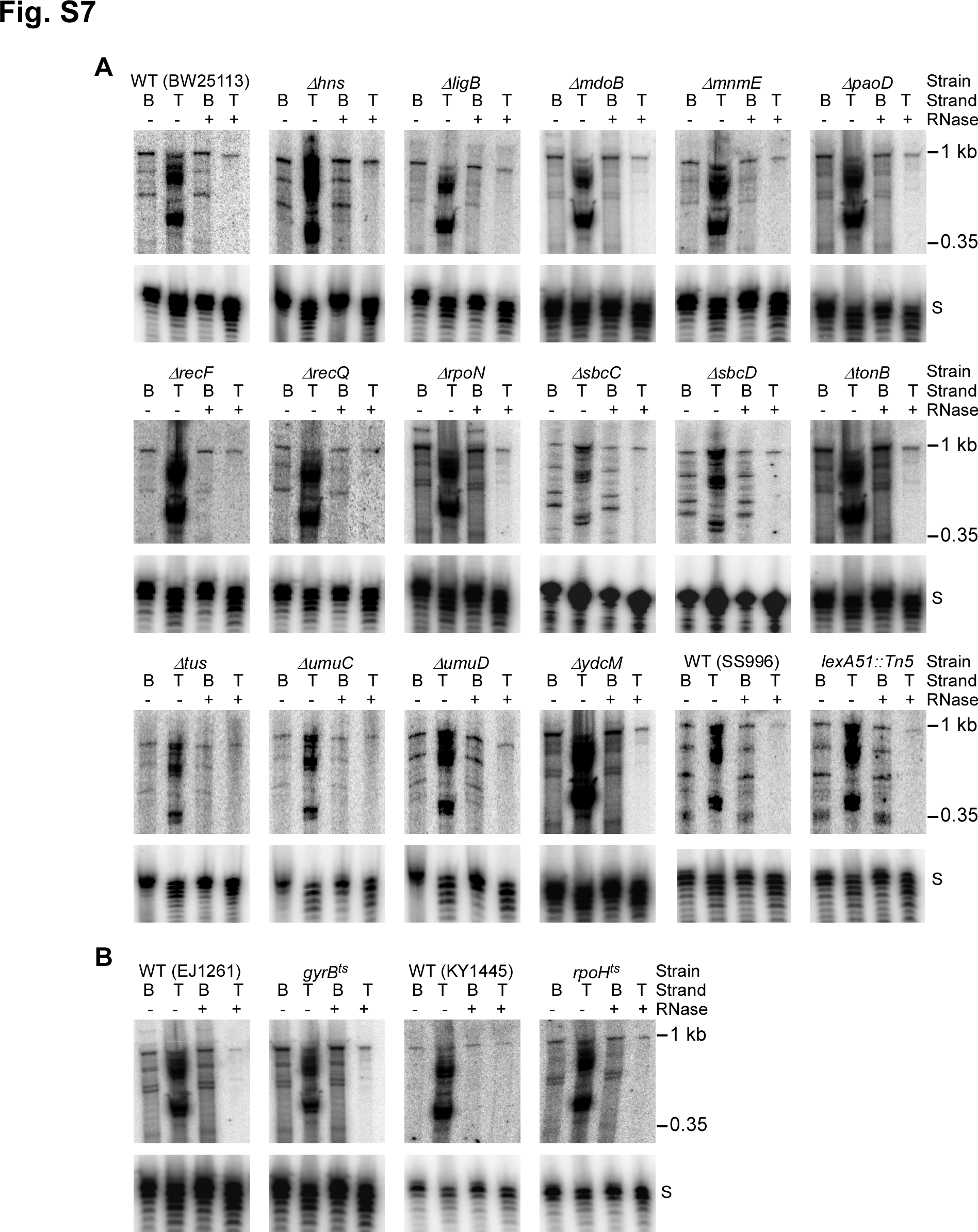

Supplement: Figure S7 — Assays of top- and bottom-strand DNA synthesis in extracts from E. coli mutant strains that did not show substantially decreased top- or bottom-strand synthesis. DNA substrates labeled at the 5′ end of either the top (T) or bottom (B) strand were incubated with group II intron RNPs for 15 min at 37°C in reaction medium containing extracts from: (A) Keio deletion mutants and their parental wild-type strain BW25113. (B) Temperature-sensitive mutants and their parental wild-type strains. After phenol-CIA extraction and proteinase K digestion, samples were split into halves that were incubated without or with RNases A+H for 30 min at 37°C. The products were analyzed in a denaturing 6% polyacrylamide gel, which was dried and scanned with a PhosphorImager. Extracts were confirmed to contain equal amounts of protein by SDS-polyacrylamide gels stained with Coomassie blue (not shown). The amount of radiolabel in the top- and bottom-strand products was normalized for the amount of substrate (S) in each lane and expressed as a percent of that in the parental wild-type strain, with results summarized in Table 2 and Table S6. At least two assays were done for each mutant and were reproducible to within <30%. (TIF) [file pgen.1003469.s007.tif]
