# Supplementary material for: Genetic and Biochemical Assays Reveal a Key Role for Replication Restart Proteins in Group II Intron Retrohoming
Source: PLoS Genet. 2013 Apr 25;9(4):e1003469. doi: 10.1371/journal.pgen.1003469 (PMC3636086; doi:10.1371/journal.pgen.1003469)
Supplement: Table S1 — Genetic assay of retrohoming efficiencies of all strains identified as TpS/GFP+ in the transposon-library screen. (DOCX) [file pgen.1003469.s008.docx]

**Table S1.** Genetic assay of retrohoming efficiencies of all strains identified as Tp^S^/GFP^+^ in the transposon-library screen.

| **Gene^a^** | **Strain** | **Insertion site^b^** | **Orientation^c^** | **Function** | **Retrohoming efficiency**  **(% WT)^d^** |
| --- | --- | --- | --- | --- | --- |
|  |  |  |  | **Transcription unit** |  |
| 1. **Nucleic acid related** | | | | | |
| *agaR* | 88H03 | 3276296 | - | Transcriptional repressor | 3.0% |
| *agaR* (promoter) | 75B11 | 3276745 | - | Transcriptional repressor | 0.2% |
| C0719 | 50G10 | 3119383 | - | Small RNA | 0.0% |
| *gyrB* / *yidB* | 97E12 | 3875600 | + | DNA gyrase, subunit B / Conserved protein | 1.1% |
| *helD* | 84D07 | 1024222 | + | DNA helicase IV | 2.7% |
| *hofB* | 34C07 | 116123 | - | Protein involved in plasmid replication  *ppdD-hofBC* | 1.6% |
| *ligB* | 65H02 | 3819167 | + | DNA ligase | 0.2% |
| *mutM* | 61F09 | 3808806 | + | Formamidopyrimidine DNA glycosylase  *rpmBG-mutM/ yicR-rpmBG-mutM* | 0.9% |
| *nudF* | 79D09 | 3175394 | - | ADP-ribose pyrophosphatase  *nudF-yqiB-cpdA-yqiA-parE* | 5.9% |
| *recJ* | 36C04 | 3035987 | + | 5’→3’ exonuclease  *dsbC-recJ-prfB/ xerD-dsbC-recJ* | 18.9% |
| *rpoH* (promoter) | 49F03 | 3598966 | + | RNA polymerase, σ^32^ (σ ^H^) factor | 3.4% |
| *rpoN* | 13F03 | 3343162 | - | RNA polymerase, σ ^54^ (σ ^N^) factor  *rpoN-hpf-ptsN-yhbJ-npr/ lptB-rpoN-hpf-ptsN-yhbJ* | 0.0% |
| *udk* | 89G08 | 2140673 | - | Uridine/cytidine kinase  *udk-dcd* | 1.6% |
| *ybeB* | 74C03 | 668116 | - | Ribosome associated protein  *ybeB-rlmH-mrdAB-rplA* | 15.2% |
| 1. **Enzymes** | | | | | |
| *aldB* (promoter) | 39G03 | 3754558 | + | Acetaldehyde dehydrogenase | 12.0% |
| *allB* | 41E03 | 539559 | + | Allantoinase  *gcl-hyi-glxR-ybbW-allB-ybbY-glxK* | 1.8% |
| *avtA* / *ysaA* | 38D08 | 3739070 | + | Valine-pyruvate aminotransferase / Predicted hydrogenase | 19.1% |
| *cadA* | 43E01 | 4355187 | + | Lysine decarboxylase I  *cadBA* | 24.5% |
| *csrD* | 38H01 | 3400305 | - | Regulator of CsrB and CsrC decay | 0.0% |
| *dsbC* | 98C09 | 3036829 | + | Protein disulfide isomerase II  *dsbC-recJ-prfB/ xerD-dsbC-recJ* | 9.8% |
| *glnD* | 45G03 | 186878 | + | Uridylyltransferase  *map-glnD-dapD* | 2.7% |
| *gpp* | 71C08 | 3961728 | - | Guanosine pentaphosphatase/exopolyphosphatase | 18.9% |
| *hslV* | 07E10 | 4119827 | - | Peptidase component of the HslUV protease  *hslVU* | 1.8% |
| *mdoB* | 18F06 | 1496373 | + | Phosphoglycerol transferase I | 8.9% |
| *mdoB* (promoter) | 45A05 | 1494782 | + | Phosphoglycerol transferase I | 3.6% |
| *metL* | 28H12 | 4130289 | - | Aspartate kinase II / Homoserine dehydrogenase II  *metBL* | 1.1% |
| *metL* | 42D11 | 4128563 | + | Aspartate kinase II / Homoserine dehydrogenase II  *metBL* | 14.1% |
| *metL* | 54F01 | 4129936 | - | Aspartate kinase II / Homoserine dehydrogenase II  *metBL* | 10.7% |
| *mhpE* | 51H09 | 373808 | + | 4-hydroxy-2-ketovalerate aldolase  *mhpABCDFE* | 2.3% |
| *mhpE* | 60E04 | 373581 | + | 4-hydroxy-2-ketovalerate aldolase  *mhpABCDFE* | 0.0% |
| *rutB* | 85C03 | 1071979 | + | Peroxyureidoacrylate / Ureidoacrylate amido hydrolase  *rutABCDEFG* | 0.0% |
| *sseA* (promoter) | 21G07 | 2650399 | - | 3-mercaptopyruvate sulfurtransferase | 3.0% |
| *waaA*(promoter) | 63D05 | 3806481 | - | KDO transferase  *waaA-coaD* | 0.7% |
| *ycaO* | 47H06 | 954715 | - | β-methylthiolation of ribosomal protein S12 | 0.0% |
| *yggF* | 70H10 | 3073259 | + | Fructose-1,6-biphosphatase  *cmtBA-yggPFDC* | 2.0% |
| *yiaK* | 05F01 | 3740962 | + | 2,3-diketo-L-gulonate dehydrogenase  *yiaKLMNO-lyxK-sgbHUE* | 0.0% |
| *yidA* | 62B11 | 3874602 | + | Sugar phosphatase | 2.5% |
| *yqiI* | 38A07 | 3188999 | - | Detoxification of methylglyoxal  *insC-5D-5-yqiGHI* | 4.8% |
| 1. **Membrane proteins** | | | | | |
| *emrE* | 87D09 | 567656 | - | Multidrug efflux transporter  *renD-emrE* | 1.1% |
| *hokC* / *nhaA* | 17D09 | 17101 | - | Toxic membrane protein / Sodium-proton antiporter  *mokC-hokC/nhaAR* | 2.3% |
| *ptsG* | 98C11 | 1158508 | + | Glucose PTS permease | 0.0% |
| *srlA* | 49A06 | 2824060 | - | Glucitol/sorbitol PTS permease  *srlAEBD-gutM-srlR-gutQ* | 1.6% |
| *tonB* | 68C08 | 1309319 | - | Membrane spanning protein | 1.1% |
| 1. **Unidentified** | | | | | |
| *htrE* | 72H04 | 154288 | - | Putative outer membrane protein  *ecpD-htrE* | 0.7% |
| *paoD* | 27G09 | 297077 | + | Conserved protein  *paoABCD* | 0.0% |
| *yaaH* | 74A07 | 10191 | - | Conserved inner membrane protein | 0.0% |
| *yagP* | 74B05 | 296606 | + | Predicted transcriptional regulator | 9.1% |
| *yahF* | 93A09 | 336215 | - | Predicted acyl-CoA synthetase  *yahDEFG* | 2.7% |
| *ybjX* | 47A12 | 917502 | - | Conserved protein | 0.0% |
| *ycjW* (promoter) | 01C07 | 1381986 | - | Predicted transcriptional regulator | 0.7% |
| *ycjZ* | 42F07 | 1390641 | - | Predicted transcriptional regulator | 2.3% |
| *ydcM* | 73C10 | 1501821 | + | Putative transposase | 6.1% |
| *yggC* | 27D12 | 3072342 | + | Conserved protein  *cmtBA-yggPFDC/yggDC* | 0.0% |
| *yghA* | 26E05 | 3147782 | - | Predicted glutathionylspermidine synthase | 6.4% |
| *ygiC* | 69B05 | 3179499 | - | Predicted enzyme  *tolC-ygiABC* | 1.6% |
| *yhdE* | 10B03 | 3395965 | - | Conserved protein | 8.2% |
| *yifO* | 03B03 | 3958084 | - | Conserved protein  *yifNO* | 6.1% |
| *yiiD* | 07F11 | 4075706 | - | Predicted acetyltransferase  *yihXY-dtd-yiiD* | 1.8% |
| *yiiD* | 41H08 | 4076177 | - | Predicted acetyltransferase  *yihXY-dtd-yiiD* | 4.3% |
| *yiiD* | 96B04 | 4075569 | - | Predicted acetyltransferase  *yihXY-dtd-yiiD* | 3.6% |
| *yjjB* | 07C10 | 4599919 | + | Conserved inner membrane protein  *yjjB-dnaTC/yjjB-dnaTC-yjjA* | 0.0% |

*E. coli* strains containing pALG3 and pBRR3-ltrB were induced with 0.5 mM IPTG for 3 h at 30°C, and the retrohoming efficiency relative to a wild-type control was determined by the plasmid-based Tp^R^-RAM assay.

^a^ Genes that were sites of transposon insertion. “Promoter” indicates insertion into the promoter region of the specified gene. "/" indicates insertion between two specified genes.

^b^ Nucleotide position of the inserted transposon numbered according to the *E. coli* K12 MG1665 genome sequence.

^c^ "+" or "-", indicate transposon insertion in the plus or minus strand of the *E. coli* chromosome.

^d^ Retrohoming efficiency determined by the 96-well plate Tp^R^-RAM assay as a percent of that for the wild-type strain assayed in parallel.
