# Supplementary material for: Genetic and Biochemical Assays Reveal a Key Role for Replication Restart Proteins in Group II Intron Retrohoming
Source: PLoS Genet. 2013 Apr 25;9(4):e1003469. doi: 10.1371/journal.pgen.1003469 (PMC3636086; doi:10.1371/journal.pgen.1003469)
Supplement: Table S2 — Taqman qPCR assays of retrohoming in other E. coli Keio deletion mutants analyzed in this work. (DOCX) [file pgen.1003469.s009.docx]

**Table S2.** Taqman qPCR assays of retrohoming in other *E. coli* Keio deletion mutants analyzed in this work.

| **Gene** | **Function** | **Retrohoming frequency**  **(% WT)** | |
| --- | --- | --- | --- |
|  |  | **5’ Junction** | **3’ Junction** |
| **(A) Nucleic acid related** | | | |
| *agaR* | Transcriptional repressor | 100 ± 3% | 121 ± 3% |
| *cpdA* | cAMP phosphodiesterase | 84 ± 9% | 92 ± 7% |
| *dtd* | D-Tyr-tRNA^Tyr^ deacylase | 81 ± 11% | 90 ± 13% |
| *helD* | DNA helicase IV | 123 ± 2% | 151 ± 15% |
| *hofB* | Protein involved in plasmid replication | 115 ± 2% | 117 ± 3% |
| *hofC* | Maintains mini-F plasmids | 110 ± 7% | 99 ± 9% |
| *mutM* | Formamidopyrimidine DNA glycosylase | 83 ± 4% | 86 ± 5% |
| *nudF* | ADP-ribose pyrophosphatase | 91 ± 6% | 89 ± 5% |
| *recO* | RecFOR recombinase component | 88 ± 10% | 94 ± 6% |
| *recR* | RecFOR recombinase component | 86 ± 7% | 89 ± 5% |
| *rpoN* | RNA polymerase σ^54^ (σ^N^) factor | 80 ± 7% | 80 ± 5% |
| *udk* | Uridine/cytidine kinase | 128 ± 4% | 116 ± 3% |
| *xerD* | Site-specific recombinase | 101 ± 3% | 84 ± 4% |
| *ybeB* | Ribosome associated protein | 137 ± 2% | 130 ± 1% |
| **(B) Enzymes** | | | |
| *aldB* | Acetaldehyde dehyrogenase | 89 ± 4% | 84 ± 5 |
| *allB* | Allantoinase | 99 ± 4% | 89 ± 6% |
| *avtA* | Valine-pyruvate aminotransferase | 139 ± 7% | 111 ± 20% |
| *cadA* | Lysine decarboxylase I | 81 ± 1% | 89 ± 2% |
| *csrD* | Regulator of csrB and csrC decay | 137 ± 13% | 137 ± 17% |
| *dsbC* | Protein disulfide isomerase II | 96 ± 8% | 91 ± 9% |
| *ecpD* | Pilin chaperone | 152 ± 5% | 154 ± 2% |
| *glnD* | Uridylyltransferase | 103 ± 2% | 108 ± 0% |
| *gpp* | Guanosine pentaphosphatase | 102 ± 7% | 96 ± 5% |
| *hslV* | Peptidase component of HslUV protease | 98 ± 8% | 91 ± 8% |
| *mdoB* | Phosphoglycerol transferase I | 73 ± 3% | 66 ± 2% |
| *metL* | Aspartate kinase II | 93 ± 4% | 92 ± 3% |
| *mhpE* | 4-hydroxy-2-ketovalerate aldolase | 84 ± 5% | 84 ± 6% |
| *rutB* | Peroxyurieidoacrylate amido hydrolase | 105 ± 8% | 100 ± 7% |
| *sseA* | 3-mercaptopyruvate sulfurtransferase | 107 ± 5% | 110 ± 8% |
| *ycaO* | β-methylthiolation of ribosomal protein S12 | 110 ± 5% | 107 ± 3% |
| *yggF* | Fructose-1,6-biphosphatase | 136 ± 7% | 129 ± 8% |
| *yiaK* | 2,3-diketo-L-gulonate dehydrogenase | 160 ± 6% | 156 ± 7% |
| *yidA* | Sugar phosphatase | 87 ± 1% | 80 ± 3% |
| *yqiI* | Detoxification of methylglyoxal | 127 ± 13% | 117 ± 13% |
| **(C) Membrane proteins** | | | |
| *emrE* | Multidrug efflux transporter | 118 ± 4% | 110 ± 6% |
| *hokC* | Toxic membrane protein | 121 ± 6% | 110 ± 4% |
| *nhaA* | Sodium-proton antiporter | 133 ± 7% | 136 ± 1% |
| *ptsG* | Glucose PTS permease | 123 ± 9% | 130 ± 1% |
| *srlA* | Glucitol/sorbitol PTS permease | 86 ± 3% | 84 ± 1% |
| *tonB* | Membrane spanning protein | 80 ± 4% | 76 ± 5% |
| *yiaM* | 2,3-diketo-L-gulonate-Na^+^ symporter subunit | 134 ± 16% | 115 ± 5% |
| *yiaN* | 2,3-diketo-L-gulonate-Na^+^ symporter subunit | 110 ± 8% | 116 ± 10% |
| *yiaO* | 2,3-diketo-L-gulonate-Na^+^ symporter subunit | 125 ± 1% | 112 ± 2% |
| **(D) Unidentified** | | | |
| *htrE* | Putative outer membrane protein | 86 ± 5% | 79 ± 6% |
| *paoD* | Conserved protein | 73 ± 2% | 68 ± 3% |
| *ppdD* | Putative type IV pilin | 121 ± 10% | 114 ± 6% |
| *yaaH* | Conserved inner membrane protein | 90 ± 5% | 100 ± 2% |
| *yagP* | Predicted transcriptional regulator | 80 ± 5% | 84 ± 4% |
| *yahF* | Predicted acyl-CoA synthetase | 85 ± 1% | 80 ± 1% |
| *ybjX* | Conserved protein | 107 ± 5% | 101 ± 5% |
| *ycjW* | Predicted transcriptional regulator | 109 ± 2% | 99 ± 1% |
| *ycjZ* | Predicted transcriptional regulator | 93 ± 7% | 89 ± 4% |
| *ydcM* | Predicted transposase | 82 ± 2% | 74 ± 1% |
| *yggC* | Conserved protein | 87 ± 7% | 96 ± 9% |
| *yggD* | Predicted transcriptional regulator | 87 ± 6% | 95 ± 6% |
| *yghA* | Predicted glutathionylspermidine synthase | 100 ± 13% | 105 ± 9% |
| *ygiC* | Predicted enzyme | 100 ± 8% | 99 ± 4% |
| *yhdE* | Conserved protein | 94 ± 10% | 88 ± 12% |
| *yidB* | Conserved protein | 94 ± 9% | 78 ± 7% |
| *yidR* | Conserved protein | 108 ± 4% | 96 ± 3% |
| *yifO* | Conserved protein | 113 ± 0% | 112 ± 9% |
| *yiiD* | Predicted acetyltransferase | 118 ± 7% | 125 ± 4% |
| *yjjB* | Conserved inner membrane protein | 89 ± 12% | 86 ± 11% |
| *ysaA* | Predicted hydrogenase | 100 ± 3% | 99 ± 3% |

The Table summarizes retrohoming frequencies for integration into a chromosomal *rhlE* target site in Keio deletion strains based on Taqman qPCR quantitation of the 5’- and 3’-integration junctions relative to the number of *rhlE* genes. Assays were done on total DNA from cells containing donor plasmid pBL1-rhlE. Cells were grown to mid-log phase at 37°C and then intron-expression induced with 4 mM *m*-toluic acid for 1 h. Values are the mean ± S.E.M. for three experimental replicates normalized to the retrohoming frequency of the wild-type control strain BW25113 assayed in parallel.
