# Supplementary material for: Genetic and Biochemical Assays Reveal a Key Role for Replication Restart Proteins in Group II Intron Retrohoming
Source: PLoS Genet. 2013 Apr 25;9(4):e1003469. doi: 10.1371/journal.pgen.1003469 (PMC3636086; doi:10.1371/journal.pgen.1003469)
Supplement: Table S3 — E. coli transposon-insertion mutants identified as TpS/GFP− in the transposon-library screen. (DOCX) [file pgen.1003469.s010.docx]

**Table S3.** *E. coli* transposon-insertion mutants identified as Tp^S^/GFP^-^ in the transposon-library screen.

| **Gene / Element^a^** | **Strain** | **Insertion site^b^** | **Orientation^c^** | **Gene product** | **Function** | **Retrohoming efficiency**  **(% WT)** |
| --- | --- | --- | --- | --- | --- | --- |
|  |  |  |  | **Transcription unit** |  |  |
| *lacUV5* | 65E06 | λ DE3 | N/A | N/A | Promoter | 0.9% |
| *lacUV5* | 66H02 | λ DE3 | N/A | N/A | Promoter | 0.8% |
| *lacUV5* | 69B02 | λ DE3 | N/A | N/A | Promoter | 1.9% |
| *lacUV5* | 70A06 | λ DE3 | N/A | N/A | Promoter | 0.8% |
| *T7RNP* | 87B02 | λ DE3 | N/A | T7 RNA polymerase | Transcription | 1.4% |
| *ugpA* | 92H03 | 3588735 | + | Glycerol-3-phosphate ABC transporter  *ugpBAECQ* | Transporter | 1.1% |
| *xylF* | 70H11 | 3729869 | + | Xylose ABC transporter subunit  *xylFGHR* | Transporter | 2.3% |
| *yjbB* | 68H09 | 4225728 | + | Putative inorganic phosphate transporter | Transporter | 2.6% |

*E. coli* strains containing intron-donor plasmid pALG3 and recipient plasmid pBRR3-ltrB were induced with 0.5 mM IPTG for 3 h at 30°C, and retrohoming efficiency relative to the wild-type strain was determined by the Tp^R^-RAM assay based on O.D._595_ measurements in 96-well plate format.

^a^ Transposon-insertion site.

^b^ Nucleotide position of the transposon insertion numbered according to the *E. coli* K12 MG1665 genome sequence or λ DE3 prophage.

^c^ "+" or "-", indicates transposon insertion in the plus or minus strand of the *E. coli* chromosome.

N/A, not applicable.
