# Supplementary material for: Genetic and Biochemical Assays Reveal a Key Role for Replication Restart Proteins in Group II Intron Retrohoming
Source: PLoS Genet. 2013 Apr 25;9(4):e1003469. doi: 10.1371/journal.pgen.1003469 (PMC3636086; doi:10.1371/journal.pgen.1003469)
Supplement: Table S4 — E. coli transposon mutants identified at GFP− in an in vivo assay in which splicing of the Ll.LtrB intron is linked to GFP expression. (DOCX) [file pgen.1003469.s011.docx]

**Table S4.** *E. coli* transposon mutants identified at GFP^-^ in an *in vivo* assay in which splicing of the Ll.LtrB intron is linked to GFP expression.

| **Gene** | **Strain^a^** | **Gene product** | **Function** |
| --- | --- | --- | --- |
|  |  | **Transcription unit** |  |
| 1. **Induction at 30°C** | | | |
| *dppD* | 46 | Dipeptide ABC transporter subunit  *dppABCDF* | Transporter |
| *kdsD* | 45 | Arabinose 5-phosphate isomerase  *yrbG-kdsD* | Enzyme |
| *malE* | 29 | Maltose ABC transporter subunit  *malEFG* | Transporter |
| *malE* | 30 | Maltose ABC transporter subunit  *malEFG* | Transporter |
| *T7RNP* | 01 | T7 RNA polymerase | Transcription |
| *T7RNP* | 21 | T7 RNA polymerase | Transcription |
| 1. **Induction at 37°C** | | | |
| *clcA* | 09 | H^+^/Cl^-^ exchange transporter | Transporter |
| *pgi / yjbE* | 61 | Phosphoglucose isomerase / Predicted protein  *yjbEFGH* | Enzyme / Unknown |
| *yfeN* | 30 | Conserved outer membrane protein | Unknown |

^a^ Mutants were identified in an assay in which *mariner* transposon library strains containing pALG2 were plated on LB agar and grown overnight at 37°C. The resulting colonies were lifted onto 35-mm diameter nitrocellulose filter circles and incubated on petripads (Millipore) saturated with LB medium containing 0.1 mM IPTG for 3 h at 30 or 37°C for induction of plasmid transcription. The filters were then examined under 302-nm UV light to detect GFP^-^ colonies.
