# Supplementary material for: Genetic and Biochemical Assays Reveal a Key Role for Replication Restart Proteins in Group II Intron Retrohoming
Source: PLoS Genet. 2013 Apr 25;9(4):e1003469. doi: 10.1371/journal.pgen.1003469 (PMC3636086; doi:10.1371/journal.pgen.1003469)
Supplement: Table S5 — E. coli mutants identified as having increased retrohoming efficiencies in the transposon-library screen. (DOCX) [file pgen.1003469.s012.docx]

**Table S5.** *E. coli* mutants identified as having increased retrohoming efficiencies in the transposon-library screen.

| **Gene^a^** | **Strain** | **Insertion site^b^** | **Orientation^c^** | **Gene product** | **Function** | **Retrohoming efficiency**  **(% WT)** |
| --- | --- | --- | --- | --- | --- | --- |
|  |  |  |  | **Transcription unit** |  |  |
| *rnlA* | 06C01 | 2764537 | - | CP4-57 prophage; RNase LS  *rnlAB* | RNA decay | 213% |
| *rnlA* | 19E08 | 2764781 | - | CP4-57 prophage; RNase LS  *rnlAB* | RNA decay | 205% |
| *rnlA* | 56G01 | 2764805 | - | CP4-57 prophage; RNase LS  *rnlAB* | RNA decay | 205% |
| *yfjK* | 53G03 | 2762631 | - | CP4-57 prophage, conserved protein  *yfjLK* | Helicase | 276% |
| *yfjL* | 22D05 | 2761349 | - | CP4-57 prophage, predicted protein  *yfjLK* | Unknown | 204% |

*E. coli* strains containing intron-donor plasmid pALG3 and recipient plasmid pBRR3-ltrB were induced with 0.5 mM IPTG for 3 h at 30°C and retrohoming efficiency relative to a wild-type control assayed in parallel was determined by the Tp^R^-RAM assay based on O.D._595_ measurements in 96-well plate format.

^a^ Transposon-insertion site.

^b^ *E. coli* chromosomal nucleotide position of the inserted transposon numbered according to the *E. coli* strain K12 MG1665 genome sequence.

^c^ "+" or "-", indicates transposon insertion in the plus or minus strand of the *E. coli* chromosome.
