# Supplementary material for: Genetic and Biochemical Assays Reveal a Key Role for Replication Restart Proteins in Group II Intron Retrohoming
Source: PLoS Genet. 2013 Apr 25;9(4):e1003469. doi: 10.1371/journal.pgen.1003469 (PMC3636086; doi:10.1371/journal.pgen.1003469)
Supplement: Table S6 — E. coli extract assays of retrohoming in wild-type and additional Keio deletion mutant strains. (DOCX) [file pgen.1003469.s013.docx]

**Table S6.** *E. coli* extract assays of retrohoming in wild-type and additional Keio deletion mutant strains.

| **Strain** | **Reverse splicing** | **Total cDNA** | **Full-length bottom strand** | **Top strand** |
| --- | --- | --- | --- | --- |
| BW25113 | 100% | 100% | 100% | 100% |
| *mdoB* | 110% | 60% | 62% | 80% |
| *paoD* | 107% | 55% | 69% | 135% |
| *rpoN* | 104% | 98% | 102% | 97% |
| *tonB* | 93% | 52% | 100% | 105% |
| *ydcM* | 149% | 53% | 49% | 95% |

Values were determined by measuring the amount of radioactivity in the indicated product band or bands relative to that in the DNA substrate band after subtraction of background and are expressed relative to the corresponding values for the parental wild-type strain assayed in parallel. Total reverse splicing was quantified by measuring the radioactivity in all bands larger than the DNA substrate band in reactions with top-strand labeled DNA substrate without RNase treatment of the products. Total cDNA was quantified by measuring the radioactivity in all bands larger than the DNA substrate band in reactions with bottom-strand labeled DNA substrate after RNase treatment of the products. Full-length bottom strand was quantified by measuring the radioactivity in the band corresponding to the full-length bottom-strand product (988 nt) in reactions with 5’ bottom-strand labeled DNA substrate after RNase treatment of the products. Full-length top strand was quantified by measuring the radioactivity in the band corresponding to the full-length top-strand product (988 nt) after RNase treatment of the products. Radioactivity was determined by scanning the dried gel with a Typhoon Trio PhosphorImager and quantifying using ImageQuant TL.
