# Supplementary material for: Genetic and Biochemical Assays Reveal a Key Role for Replication Restart Proteins in Group II Intron Retrohoming
Source: PLoS Genet. 2013 Apr 25;9(4):e1003469. doi: 10.1371/journal.pgen.1003469 (PMC3636086; doi:10.1371/journal.pgen.1003469)
Supplement: Table S7 — E. coli strains used in this work. (DOCX) [file pgen.1003469.s014.docx]

**Table S7.** *E. coli* strains used in this work.

| **Strain** | ***ts*** | **Genotype** |
| --- | --- | --- |
| AB1157^a^ | N | F^-^, *thr-1*, *araC14*, *leuB6*(Am), *Δ(gpt-proA)62*, *lacY1*, *tsx-33*, *qsr'-0*, *glnV44*(AS), *galK2*(Oc), l*^-^*, *Rac-0*, *isG4*(Oc), *rfbC1*, *mgl-51*, *rpoS396*(Am), *rpsL31*(strR), *kdgK51*, *xylA5*, *mtl-1*, *argE3*(Oc), *thi-1* |
| AB1157*dnaE^ts^* | Y | F^-^, *thr-1*, *araC14*, *leuB6*(Am), *Δ(gpt-proA)62*, *lacY1*, *tsx-33*, *qsr'-0*, *glnV44*(AS), *galK2*(Oc), l*^-^*, *Rac-0*, *isG4*(Oc), *rfbC1*, m*gl-51*, *rpoS396*(Am), *rpsL31*(strR), *kdgK51*, *xylA5*, *mtl-1*, *argE3*(Oc), *thi-1,dnaE486*(ts), *zae502::Tn10* |
| BW25113^b^ | N | F^-^, *Δ(araD-araB)567*, *ΔlacZ4787*(::rrnB-3), *λ^-^*, *rph-1*, *Δ(rhaD-rhaB)568*, *hsdR514* |
| BW30384^c^ | N | F^-^, *l^-^*, *IN(rrnD-rrnE)1* |
| DG76^d^ | N | F^-^, *leuB6*(Am), *l^-^*, *thyA47,* *rpsL153*(strR), *deoC3* |
| DH5α | N | F^-^, φ80*lacZΔ*M15 *Δ*(*lacZYA*-*argF*), U169, *recA1*, *endA1*, *hsdR17*(r_K_^-^, m_K_^+^), *gal*^-^, *phoA*, *supE44*, λ^-^, *thi*-*1*, *gyrA96*, *relA1* |
| EJ1261^e^ | N | F^-^, *galK2*(Oc), *λ^-^*, *IN(rrnD-rrnE)1*, *rpsL200*(strR), *maeA1* |
| HMS174(DE3) | N | F^-^, *recA1*, *hsdR*(r_K12_^-^, m_K12_^+^), *Rif*^r^, λDE3 |
| KL921^f^ | N | F^-^, *Δ(gpt-lac)5*, *LAMcI*(ind), *thyA0*, *rpsL-*(strR), *malE145::Tn10*, *deo-* |
| KL922*ssb^ts^* | Y | F^-^, *Δ(gpt-lac)5*, *LAMcI*(ind), *thyA0*, *rpsL-*(strR), *malE145::Tn10*, *deo-,ssb-1*(ts) |
| KY1429*rpoH^ts^* | Y | F^-^, *[araD139]_B/r_*, *Δ(argF-lac)169*, *λ^-^*, *flhD5301*, *Δ(fruK-yeiR)725(fruA25)*, *relA1*, *rpsL150*(strR), *zhh-50::Tn10*, *rpoH606*(ts), *rbsR22*, *Δ(fimB-fimE)632(::IS1)*, *deoC1* |
| KY1445^g^ | N | F^-^, *[araD139]_B/r_*, *Δ(argF-lac)169*, *λ^-^*, *flhD5301*, *Δ(fruK-yeiR)725(fruA25)*, *relA1*, *rpsL150*(strR), *zhh-50::Tn10*, *rbsR22*, *Δ(fimB-fimE)632(::IS1)*, *deoC1* |
| N2603^h^ | N | F^-^, *strA^r^, ptsI105, r^-^m^+^, gal^+^* |
| N2603*ligA^ts^* | Y | F^-^, *strA^r^, ptsI105, r^-^m^+^, gal^+^,lig7*(ts) |
| N4177*gyrB^ts^* | Y | F^-^, *galK2*(Oc), *λ^-^*, *IN(rrnD-rrnE)1*, *rpsL200*(strR), *gyrB221*(Cou^R^), *gyrB203*(ts) |
| PC1*dnaC^ts^* | Y | F^-^, *leuB6*(Am), *l^-^*, *thyA47,* *rpsL153*(strR), *deoC3,dnaC1*(ts) |
| PC3*dnaG^ts^* | Y | F^-^, *leuB6*(Am), *l^-^*, *thyA47,* *rpsL153*(strR), *deoC3,dnaG3*(ts) |
| PC8*dnaB^ts^* | Y | F^-^, *leuB6*(Am), *l^-^*, *thyA47,* *rpsL153*(strR), *deoC3,dnaB8*(ts) |
| PR7 | N | F-, *thr-1*, *leuB6*(Am), *lacY1*, *rna-19*, *λ^-^*, *pnp-7*, *rpsL132*(strR), *malT1*(λ^R^), *xyl-7*, *mtlA2*, *thiE1* |
| PR100^i^ | N | F-, *thr-1*, *leuB6*(Am), *lacY1*, *rna-19*, *λ^-^*, *rpsL132*(strR), *malT1*(λ^R^), *xyl-7*, *mtlA2*, *thiE1* |
| RS5064*polAex^ts^* | Y | F^-^*, l^-^,trpA33*, *IN(rrnD-rrnE)1*, *polA480*(ts,EX) |
| SS996^j^ | N | F*^-^*, lacMS286, *argE3*, *his-4*, *thi-1*, *xyl-5*, *mtl-1*, *sulB103*, *Δ* (attB):P*sulA*-*gfp* |
| SS1091 | N | F*^-^*, lacMS286, *argE3*, *his-4*, *thi-1*, *xyl-5*, *mtl-1*, *sulB103*, *Δ* (attB):P*sulA*-*gfp*, *dnaC809* |
| SS1411 | N | F*^-^*, lacMS286, *argE3*, *his-4*, *thi-1*, *xyl-5*, *mtl-1*, *sulB103*, *Δ* (attB):P*sulA*-*gfp*, *priA2::kan* |
| SS1419 | N | F*^-^*, lacMS286, *argE3*, *his-4*, *thi-1*, *xyl-5*, *mtl-1*, *sulB103*, *Δ* (attB):P*sulA*-*gfp*, *zji-202::Tn10*, *dnaT822* |
| SS1443 | N | F*^-^*, lacMS286, *argE3*, *his-4*, *thi-1*, *xyl-5*, *mtl-1*, *sulB103*, *Δ* (attB):P*sulA*-*gfp*, *Δ* (*priB*)*302* |
| SS3403 | N | F*^-^*, lacMS286, *argE3*, *his-4*, *thi-1*, *xyl-5*, *mtl-1*, *sulB103*, *Δ* (attB):P*sulA*-*gfp*, *priC303::kan* |
| SS4610 | N | F*^-^*, lacMS286, *argE3*, *his-4*, *thi-1*, *xyl-5*, *mtl-1*, *sulB103*, *Δ* (attB):P*sulA*-*gfp*, *lexA51::Tn5* |
| SS6239 | N | F*^-^*, lacMS286, *argE3*, *his-4*, *thi-1*, *xyl-5*, *mtl-1*, *sulB103*, *Δ* (attB):P*sulA*-*gfp*, *zae-502::Tn10*, *dnaE486* |
| SS6253 | N | F*^-^*, lacMS286, *argE3*, *his-4*, *thi-1*, *xyl-5*, *mtl-1*, *sulB103*, *Δ* (attB):P*sulA*-*gfp*, *malF::Tn10*, *dnaB8*(ts) |
| SS6668 | N | F*^-^*, lacMS286, *argE3*, *his-4*, *thi-1*, *xyl-5*, *mtl-1*, *sulB103*, *Δ* (attB):P*sulA*-*gfp*, *aer-3075::Tn10*, *dnaG2903* |

*^ts^* Temperature sensitive.

^a^ AB1157 is the parental strain of AB1157*dnaE^ts^*, from Dr. Marlene Belfort (University at Albany, State University of New York).

^b^ BW25113 is the parental strain of the Keio collection, from the National BioResource Project (NIG, Japan).

^c^: BW30384 is the parental strain of RS5064*polAex^ts^*, from the Coli Genetic Stock Center (CGCS) at Yale.

^d^: DG76 is the parental strain of PC1*dnaC^ts^*, PC3*dnaG^ts^*, and PC8*dnaB^ts^*, from CGCS.

^e^: EJ1261 is the parental strain of N4177*gyrB^ts^*, from CGCS.

^f^: KL921 is the parental strain of KL922*ssb^ts^*, from CGCS.

^g^: KY1445 is the parental strain of KY1429*rpoH^ts^*, from CGCS.

^h^: N2603 is the parental strain of N2603*ligA^ts^*, from Dr. Marlene Belfort (University at Albany, State University of New York).

^i^: PR100 is the parental strain of PR7, from CGCS.

^j^: SS996 is the parental strain of other SS strains, from Dr. Steven Sandler (University of Massachusetts).
